# Genomic Comparison of *Escherichia coli* O104:H4 Isolates from 2009 and 2011 Reveals Plasmid, and Prophage Heterogeneity, Including Shiga Toxin Encoding Phage stx2

**DOI:** 10.1371/journal.pone.0048228

**Published:** 2012-11-01

**Authors:** Sanaa A. Ahmed, Joy Awosika, Carson Baldwin, Kimberly A. Bishop-Lilly, Biswajit Biswas, Stacey Broomall, Patrick S. G. Chain, Olga Chertkov, Otar Chokoshvili, Susan Coyne, Karen Davenport, J. Chris Detter, William Dorman, Tracy H. Erkkila, Jason P. Folster, Kenneth G. Frey, Matroner George, Cheryl Gleasner, Matthew Henry, Karen K. Hill, Kyle Hubbard, Joseph Insalaco, Shannon Johnson, Aaron Kitzmiller, Michael Krepps, Chien-Chi Lo, Truong Luu, Lauren A. McNew, Timothy Minogue, Christine A. Munk, Brian Osborne, Mohit Patel, Krista G. Reitenga, C. Nicole Rosenzweig, April Shea, Xiaohong Shen, Nancy Strockbine, Cheryl Tarr, Hazuki Teshima, Eric van Gieson, Kathleen Verratti, Mark Wolcott, Gary Xie, Shanmuga Sozhamannan, Henry S. Gibbons, Threat Characterization Consortium

**Affiliations:** 1 Los Alamos National Laboratory, Los Alamos, New Mexico, United States of America; 2 Naval Medical Research Center and Henry M. Jackson Foundation for Military Medicine, Frederick, Maryland, United States of America; 3 United States Army Research Institute for Infectious Disease, Frederick, Maryland, United States of America; 4 United States Army Edgewood Chemical Biological Center, Aberdeen Proving Ground, Maryland, United States of America; 5 South Caucasus Field Epidemiology and Laboratory Training Program, National Center for Disease Control and Public Health, Tbilisi, Republic of Georgia; 6 Enteric Diseases Laboratory Branch, Centers for Disease Control and Prevention, Atlanta, Georgia, United States of America; 7 Excet Inc, Springfield, Virginia, United States of America; 8 Science Applications International Corporation, Abingdon, Maryland, United States of America; 9 BioTeam, Inc., Middleton, Massachusetts, United States of America; 10 Team Ke’aki Tech, Frederick, Maryland, United States of America; 11 Defense Threat Reduction Agency, Alexandria, Virginia, United States of America; U. S. Salinity Lab, United States of America

## Abstract

In May of 2011, an enteroaggregative *Escherichia coli* O104:H4 strain that had acquired a Shiga toxin 2-converting phage caused a large outbreak of bloody diarrhea in Europe which was notable for its high prevalence of hemolytic uremic syndrome cases. Several studies have described the genomic inventory and phylogenies of strains associated with the outbreak and a collection of historical *E. coli* O104:H4 isolates using draft genome assemblies. We present the complete, closed genome sequences of an isolate from the 2011 outbreak (2011C–3493) and two isolates from cases of bloody diarrhea that occurred in the Republic of Georgia in 2009 (2009EL–2050 and 2009EL–2071). Comparative genome analysis indicates that, while the Georgian strains are the nearest neighbors to the 2011 outbreak isolates sequenced to date, structural and nucleotide-level differences are evident in the Stx2 phage genomes, the *mer/tet* antibiotic resistance island, and in the prophage and plasmid profiles of the strains, including a previously undescribed plasmid with homology to the pMT virulence plasmid of *Yersinia pestis*. In addition, multiphenotype analysis showed that 2009EL–2071 possessed higher resistance to polymyxin and membrane-disrupting agents. Finally, we show evidence by electron microscopy of the presence of a common phage morphotype among the European and Georgian strains and a second phage morphotype among the Georgian strains. The presence of at least two stx2 phage genotypes in host genetic backgrounds that may derive from a recent common ancestor of the 2011 outbreak isolates indicates that the emergence of stx2 phage-containing *E. coli* O104:H4 strains probably occurred more than once, or that the current outbreak isolates may be the result of a recent transfer of a new stx2 phage element into a pre-existing stx2-positive genetic background.

## Introduction

Pathogenic *Escherichia coli* strains are capable of causing a number of disease states in humans and animals and colonizing a variety of niches within these hosts [1]. The ability of certain pathotypes of *E. coli* to colonize agriculturally important domestic animals and survive in meat products makes these organisms a particularly common cause of foodborne infections [2], [3]. In addition some *E. coli* strains have been shown to colonize plant tissues following contamination of soils or irrigation water from infected herds or wildlife, resulting in large outbreaks that have been attributed to sprouts or contaminated vegetables [4], [5], [6], [7], [8]. In the case of enterohemorrhagic *E. coli* (EHEC) strains that produce Shiga toxins (Stx), infection of a susceptible host results in fever and bloody diarrhea, and can progress in some cases to hemolytic uremic syndrome (HUS) and other severe complications, which can be fatal [9], [10]. Because of their relatively high pathogenicity and ease of transmission, pathogenic *E. coli* strains have been classified as potential agents of bioterrorism [11].

**Table 1:** Strains and their sources.

Strain	Description	Source	Reference
2011C–3493	*Escherichia coli* O104:H4, positive for *stx2a*,*aggR, aatA,* and fermentation of sorbitol, lactoseand beta-glucuronidase. Negative for *eae*and enterohemolysin production(*ehxA*).CTX15M-positive and Cef^R^	U.S. Centers for Disease Control, 2011	This work
2009EL–2050	*Escherichia coli* O104:H4, positive for *stx2a*,*aggR, aatA,* and fermentation of sorbitol, lactoseand beta-glucuronidase. Negative for *eae*and enterohemolysin production(*ehxA*). Cef^S^	National Centers for Disease Controland Public Health, Tbilisi, Republic ofGeorgia, 2009	[30]
2009EL–2071	*Escherichia coli* O104:H4, positive for *stx2a*,*aggR, aatA,* and fermentation of sorbitol, lactoseand beta-glucuronidase. Negative for *eae*and enterohemolysin production (*ehxA*). Cef^S^	National Centers for Disease Controland Public Health, Tbilisi, Republic ofGeorgia, 2009	[30]

Human pathogenic *E. coli* strains exhibit a wide spectrum of phenotypes and clinical manifestations and can colonize a broad range of tissues and body sites. The tissue tropism for any given strain is largely dependent on the genetic armamentarium that each strain possesses. Strains can vary dramatically in their genetic complement; with a variety of exchangeable elements, including plasmids, transposons, pathogenicity islands, and other mobile elements, most notably cryptic, active, and lysogenic bacteriophage (reviewed in ref. [1]). These elements can, separately or together, carry elements encoding antibiotic resistance; bacterial toxins; extracellular structures promoting adhesion (fimbriae, pili); and the extracellular polysaccharide and flagellar subunits that designate their serotypes (e.g. O157:H7) [12], [13]. New combinations of these chromosomal and extrachromosomal elements continually emerge and propagate in the environment and in susceptible hosts, leading to host shifts and new clinical presentations.

During the last two decades, most reported incidents of HUS have been attributed to *E. coli* strains belonging to serotype O157:H7 [14]. However, in the past few years diagnostic tests targeting Shiga toxins have allowed detection of Shiga toxin-producing *E. coli* (STEC) strains belonging to different serotypes from cases of hemorrhagic colitis and HUS [15]. Genes encoding Stx and Stx variants are located on transmissible prophages that are carried in the chromosomes of each strain. Stx-encoding prophage can excise and begin replicating when the bacteria are subjected to DNA-damaging growth conditions including the presence of antibiotics [16], [17], [18], and phage particles arising from such events can result in horizontal transmission of the Shiga toxin genes through the lysogeny of unrelated *E. coli* strains [19]. The Stx toxins themselves are thought to mediate the most severe consequences of STEC infection by causing toxicity and inflammation of the kidneys [9], [20].

In contrast to classical EHEC strains, which colonize the intestine by means of an elaborate Type III secretion system encoded on a pathogenicity island [21] that facilitates the actin-mediated formation of pedestals on host cell surfaces to which the colonizing bacteria adhere [22], enteroaggregative strains (EAggEC) exhibit dramatically different strategies for colonization and infection. During colonization of the colon and ileum, these strains express enteroaggregative fimbriae [23], [24] and form dense biofilm-like aggregates that adhere tightly to the epithelial layer. These aggregates are rendered flexible by the expression of dispersin, which also aids penetrance of mucous layers, EAggEC strains express a repertoire of lineage-specific virulence factors which includes SPATE proteins, (serine protease autotransporter protein of Enterobacteriaceae; [25]) including the mucinolytic Pic protein [26], and more strain-specific toxins including Pet [27]. Like other pathogenic E. coli strains, EAggEC strains are susceptible to infection by lambdoid phages including stx phages: a report of a 2001 case of HUS caused by a *stx2*-positive EAggEC belonging to the O104:H4 serotype [28], [29] was followed in 2009 by several cases of HUS and bloody diarrhea cases in the Republic of Georgia, which were eventually attributed to strains of *stx2*-positive*E. coli* O104:H4 strains (ref. [30] and Chokoshvili O. *et al.*, manuscript in preparation). Simultaneously with the work presented in this study, Beutin et al undertook an independent characterization limited to the stx2 prophages present in those 2009 strains [31].

**Table 2:** Strain Characteristics and Antibiotic Resistance Profiles.

	2009, Republic of Georgia		2011, Western Europe
CDC #	2009EL–2050	2009EL–2071	2011C–3493
**Travel to Germany**	N/A	N/A	Yes
**Patient Sex**	ND	ND	Male
**Patient Age**	ND	ND	51
**Origin**	Human	Human	Human
**Source**	stool	stool	stool
**Illness**	bloody diarrhea	bloody diarrhea	HUS
**Date of Illness Onset**	2009	2009	5/18/2011
**Date of Collection**	2009	2009	5/25/2011
**O-Antigen**	104	104	104
**H-Antigen**	4	4	4
**PFGE XbaI Pattern**	EXAX01.0002	EXAX01.0001	EXAX01.0003
**PFGE BlnI Pattern**	EXAA26.0002	EXAA26.0001	EXAA26.0003
***stx1***	–	–	–
***stx2***	+	+	+
***eae***	–	–	–
***ehxA***	–	–	–
***stx2*** ** Subtype**	*stx2a*	*stx2a*	*stx2a*
***aatA***	+	+	+
***aggR***	+	+	+
***ipaH***	–	–	–
**LT**	–	–	–
**STh**	–	–	**–**
**STp**	–	–	**–**
**Shiga Toxin Titer** 2	25	25	5
**Amoxicillin/Clavulanic Acid**	S^3^ (4)	S (8)	S (8)
**Ampicillin**	R (>32)	R (>32)	R (>32)
**Azithromycin**	NI (4)	NI (2)	NI (4)
**Cefoxitin**	S (4)	S (2)	S (2)
**Ceftiofur**	S (0.25)	S (0.25)	R (>8)
**Ceftriaxone**	S (< = 0.25)	S (< = 0.25)	R (>64)
**Chloramphenicol**	S (4)	S (4)	S (4)
**Ciprofloxacin**	S (0.06)	S (0.06)	S (0.06)
**Gentamicin**	S (0.5)	S (0.5)	S (0.5)
**Kanamycin**	S (< = 8)	S (< = 8)	S (< = 8)
**Nalidixic Acid**	S (16)	S (16)	S* (16)
**Streptomycin**	R (>64)	R (>64)	R (>64)
**Sulfisoxazole**	R (>256)	R (>256)	R (>256)
**Tetracycline**	R (32)	S (< = 4)	R (32)
**Trimothoprim/Sulphamethoxazole**	R (>4)	R (>4)	R (>4)

1 All strains were isolated from human stool and were positive by PCR for *stx2a*, *aatA* and *aggR.* The genes for *stx1*, *eae*, and *ehxA* were not detected by PCR.

2 Determined by ELISA (Premier EHEC, Meridian Biosciences, Inc. Cincinati, OH). Titer represencents the reciprocal of the highest dilution of the sample (enrichment broth) that yielded a positive reaction according to the manufacturer’s instruction.

3 Interpretive criteria to categorize minimum inhibitory concentration results as susceptible, intermediate or resistant are based on current guidelines provided by the Clinical and Laboratory Standards Institute (CLSI). Interpretive criteria published in the most recent National Antimicrobial Resistance Monitoring System (NARMS) annual report (www.cdc.gov/narms) are applied for drugs that lack CLSI interpretive criteria.

Strains of the O104:H4 serotype harboring the Stx2-encoding phage received little notice until a *stx2*-positive EAggEC (StxEAggEC) strain caused a large outbreak centered in Germany [30], [32] and a small outbreak in France [33]. In all, 16 countries in Europe and North America reported a total of 4075 cases and 50 deaths. Hemolytic-uremic syndrome (HUS) was a frequent complication of the illness in these outbreaks, occurring in 22% of the reported illnesses [34]. The source of the O104:H4 infection in Germany and France was traced to sprouts derived from fenugreek seeds that had been imported into Europe from Egypt in 2009 [9], [10], [35]. Early results indicated that the 2011 outbreak strain of *E. coli* O104:H4, while expressing the Shiga toxin typical of enterohemorrhagic (EHEC) strains, shared features of enteroaggregative (EAggEC or EAEC) strains [32], [36]. While the strain was characterized rapidly by a series of efforts spread across the globe [29], [36], [37], [38], no closed, finished sequence of the outbreak strain was presented. Furthermore, the origins and evolutionary history of the 2011 outbreak strain remain obscure.

**Figure 1:**
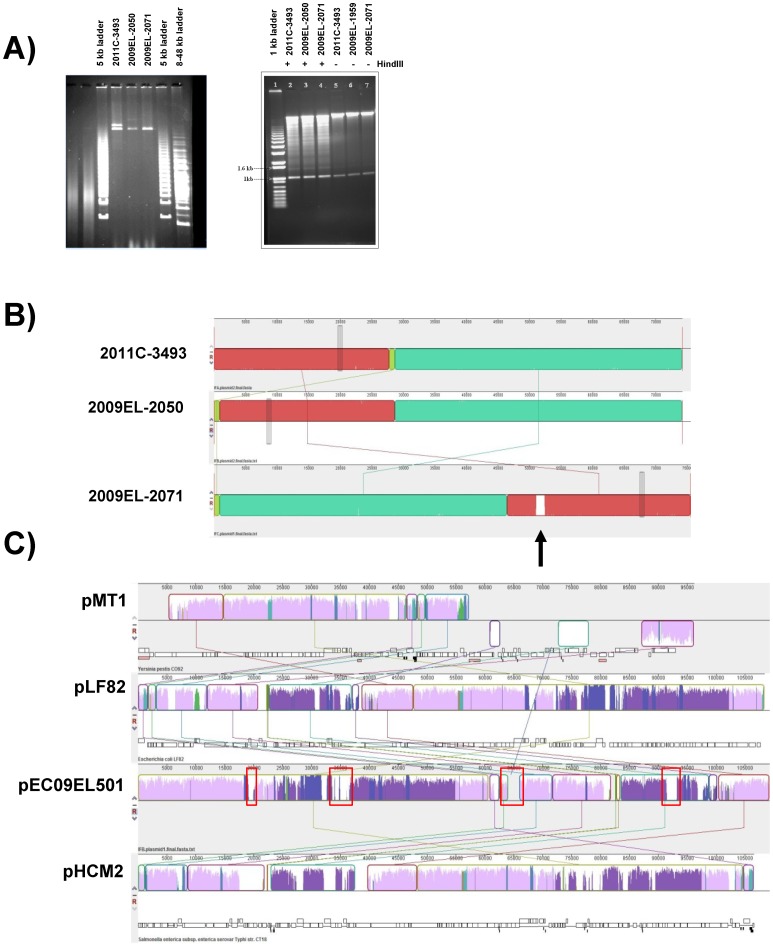
Georgian strains and 2011 Outbreak Isolates have Different Plasmid Profiles. A) Plasmid profiles of Georgian and 2011 Outbreak isolates. Left panel: PFGE of high-molecular weight plasmids. Right panel: conventional agarose gels showing small, *Hin*DIII-resistant 1.5 kb plasmids. **B)** Structure of pAA-09EL50pAA-09EL50 and pAA-09EL71 and comparison to pAA/pAA-EA11 by MAUVE. The sequences are nearly identical but for the presence of IS element-associated sequence in pAA-09EL71 (arrow). **C)** Comparison of p09EL50 to the 108kb plasmid pLF82 from *E. coli* LF82 [62], *Salmonella enteric* subsp. *enterica* serovar Typhi *typhi* str. CT18 plasmid pHCM2, and *Yersinia pestis* CO92 plasmid pMT1. Elements common to all three plasmids are colored in light purple, whereas elements lacking in one or more strains are colored in dark purple.

We obtained isolates of *E. coli* O104:H4 strains from a previous case cluster in the Republic of Georgia in 2009 and compared them to a representative isolate of the 2011 outbreak strain. The isolate from the 2011 outbreak was obtained from a patient hospitalized in the United States who had travelled to the outbreak zone in Germany in May. To characterize the strain, we performed both multi-phenotypic analysis and whole-genome sequencing of each isolate using both classical and high-throughput molecular approaches. We present the first fully closed, finished genome sequences of each isolate and compare the genetic content of each strain including but not limited to the prophage regions. The 2011 outbreak strain was distinguishable from the 2009 strains by the presence of a plasmid encoding a CTX15 beta-lactamase and by differences in the prophage content, chromosomal and plasmid sequences, and the profiles of mobile genetic element insertions. Despite a common and very closely related core genome, each strain carried a distinct repertoire of unique genomic and plasmid regions, with particular divergence in the bacteriophage loci encoded in each genome. Furthermore we show induction of at least two distinct bacteriophages from two of the three strains. The phenotypic differences and molecular diversity of Shiga toxin*-*positive EAggEC suggest that significant uncharacterized diversity exists within this clade of *E. coli* strains that may pose additional outbreak risks.

## Methods

### Strains Examined in this Study and from Previously Published Studies

All strains were isolated from human stool. Strain 2011C–3493 was isolated from a US patient with a history of travel to Germany in May 2011and strains 2009EL–2050 and 2009EL–2071 were isolated each from different patients in the Republic of Georgia (Table 1). Because the bacterial isolates used in this study are publicly available and non-identifiable, the work conducted with these isolates does not involve human subjects, as defined in the existing U.S. Federal regulations for human subject research (see 45 CFR 46.102(f)). Informed consent was not obtained since these isolates were collected in the course of routine patient care; secondary use of such non-identifiable isolates does not require informed consent per human subjects protection regulations.

The strains from this study were compared to the following *E. coli* O104:H4 strains previously described in the literature: strain TY2482 (*stx2*+ EAggEC from a case of bloody diarrhea in a 16 year old girl from Germany, 2011) [38], 55989 (*stx*-negative EAggEC from a case of persistent watery diarrhea in an HIV-infected adult from the Central African Republic, 2001) [38], [39] and HUSEC041 (01–09591) (*stx2*+ EAggEC from a case of HUS in Germany,in 2001) [28], [29].

**Table 3:** Comparison of plasmid sequences to databases.

Strain						
TY2482	2011C–3493	2009EL–2050	2009EL–2071	HUSEC041	Closest homolog	Note
pG TY2482	pG-EA11	pG-09EL50	pG-09EL71		gb|JF813186.1| *Shigella flexneri* strain 2a 301 plasmid pSF301-1; Length = 1549	Cryptic plasmids, only encode repA genes
pESBL	pESBL-EA11				gb|GU371927.1| *Escherichia coli* plasmid pEC_Bactec; Length = 92970	IncI1 plasmid, carries CTX-M-15 beta-lactamase
pAA	pAA-EA11	pAA-09EL50	pAA-09EL71	pHUSEC041–2; (73.6 kb)	emb|CU928159.2| *Escherichia coli* str. 55989 plasmid 55989p; Length = 72482	Poor alignment, a Tn5-insert derivative of adherence plasmid
		p09EL50			emb|CU638872.1| *Escherichia coli* LF82 plasmid; Length = 108379	Also similar to plasmids from *Yersinia pestis* (pMT1) and *Salmonella typhimurium* (pHCM1).
				pHUSEC041–3 (7.93 kb)	gb|EU580135.1| *Escherichia coli* strain E2348/69 plasmid p5217, complete sequence; Length = 5217	Cryptic plasmid, encoded rop and several mobilization genes. pHUSEC041-3 has no matching with anything after 3560bp
				pHUSEC041–1 (91.9 kb)	gb|GU256641.1|*Escherichia coli* plasmid p3521; Length = 110416	IncB plasmid from *Escherichia coli* encoding ACC-4, SCO-1, and TEM-1 beta-lactamases.
				pHUSEC041–4 (5.1 kb)	gb|CP000642.1| *Shigella sonnei* Ss046 plasmid pSS046_spB, complete sequence; Length = 5153	

### Antimicrobial Susceptibility Testing

Broth microdilution (Sensititre®, Trek Diagnostics, Westlake, OH) was used to determine the minimum inhibitory concentrations (MIC) for 15 antimicrobial agents; amikacin, ampicillin, amoxicillin-clavulanic acid, cefoxitin, ceftiofur, ceftriaxone, chloramphenicol, ciprofloxacin, gentamicin, kanamycin, nalidixic acid, streptomycin, sulfisoxazole, tetracycline, and trimethoprim-sulfamethoxazole. Resistance was defined by the Clinical and Laboratory Standards Institute (CLSI) interpretive standards, when available (CLSI (2011). “Performance Standards for Antimicrobial Susceptibility Testing; Twenty-first Informational Supplement. CLSI Document M100-S21.” Clinical and Laboratory Standards Institute). For streptomycin, where no CLSI interpretive criteria for human isolates exist, the resistance breakpoint is 64 µg/ml (Centers for Disease Control and Prevention (2009). “National Antimicrobial Resistance Monitoring System for Enteric Bacteria (NARMS): Enteric Bacteria Annual Report). Testing was performed according to the manufacturer’s instructions and the following quality control strains; *E. coli* ATCC 25922, *Staphylococcus aureus* ATCC 29213, *E. coli* ATCC 35218, and *Pseudomonas aeruginosa* ATCC 27853.

**Figure 2:**
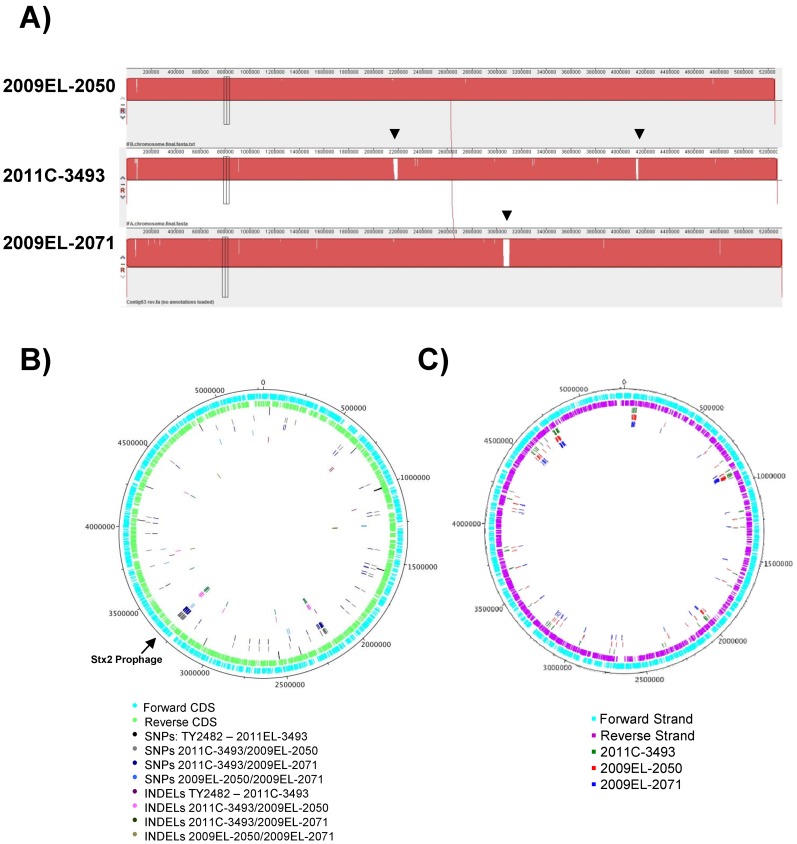
The chromosomal architecture of 2009 and 2011 strains is similar. A) Alignment of the chromosomes of the 2011 (2011C–3493) and 2009 outbreak genomes (2009EL–2050, 2009EL–2071) in MAUVE. Large regions of divergence are shown as white gaps. **B)** Locations of SNPs and small insertion/deletions in the 2009 and 2011 genomes. Most of the apparent differences between TY2482 and 2011EL–3493 in this map are due to sequence errors in the TY2482 sequence [65]; see Table S4. **C)** Location of insertion sequences (IS) and IS-like elements.

### Plasmid DNA Isolation

To visualize large plasmids present in the *E. coli* strains characterized here, the following protocol was used to isolate intact plasmids. Bacterial cultures were streaked out on Luria-Bertani (LB) agar plates from −70°C stocks and plates were incubated at 37°C for 18 hours for colony formation. Overnight cultures were prepared from isolated colonies grown on agar plates. Briefly, 20 ml of LB broth was inoculated with 3 to 4 isolated bacterial colonies and incubated at 37°C for 20 hours with shaking at 225 rpm. Three ml of each bacterial culture were centrifuged at 12,000×g for 2 minutes at room temperature, the supernatants were removed and the cell pellets were thoroughly suspended in 250 µl of an ice cold solution of 50 mM glucose, 10 mM EDTA, and 25 mM Tris-HCL, pH 8.0. All sample preparations were handled gently during and after lysis to prevent shearing of the supercoiled DNA. The tubes were incubated at room temperature for 5 minutes prior to addition of 250 µl of a solution of 0.2 N NaOH and 1% SDS to lyse the cells followed by gentle mixing by inverting the tube six times and holding at room temperature for 5 minutes. After lysis, 3 M potassium acetate solution pH 4.8 (250 µl) was added to a concentration of 1 M, and the lysates were mixed by gently inverting the tubes 10 times followed by centrifugation at 12,000×g for 5 minutes at room temperature. The supernatants were transferred to fresh 1.5 ml tubes, centrifuged another 5 minutes to avoid carryover of any precipitated material and the supernatants were transferred to new tubes. The cleared supernatants were treated with RNase A to a final concentration of 50 ug/ml and incubated at 37°C for 30 minutes. After RNase treatment, supernatants were extracted twice with phenol:chloroform and 3 times with chloroform:isoamyl alcohol. The nucleic acids from supernatants were precipitated by addition of 0.1 volume of 3 M sodium acetate (pH 5.0) and 100% ethanol followed by incubation at −20°C for an hour and centrifugation at 15,000×g for 30 minutes at 4°C. The pellets were rinsed with 1 ml of ice cold 70% ethanol and centrifuged as described above. The supernatants were discarded and the pellets were dried under vacuum without applying heat. The pellets were hydrated with 100 µl of distilled water and stored at 4°C overnight to rehydrate the DNA. DNA samples were concentrated to about 5 fold (∼20 µl) under vacuum before loading onto an agarose gel for pulsed field gel electrophoretic analysis.

**Table 4:** SNPs, Indels and Large Gaps.

		SNPs						
Reference	Query	Total	Intergenic	Synonymous	Non-synonymous	INDELs	Gap	Gap_bases
TY2482_chromosome	3493	29	6	14	9	6	7	859
pESBL	3493	16	16	0	0	0	3	104
pAA	3493	0	0	0	0	0	0	0
pG TY2482	3493	0	0	0	0	0	0	0
TY2482_chromosome	2050	262	61	132	69	19	23	41021
pESBL	2050	N/A	N/A	N/A	N/A	N/A	N/A	N/A
pAA	2050	1	0	1	0	0	0	0
pG TY2482	2050	0	0	0	0	0	1	20
TY2482_chromosome	2071	263	54	131	78	18	19	34299
pESBL	2071	N/A	N/A	N/A	N/A	N/A	N/A	N/A
pAA	2071	1	0	1	0	0	0	0
pG TY2482	2071	0	0	0	0	0	0	0
3493_chromosome	2050	262	61	127	74	16	19	40965
pESBL-EA11	2050	N/A	N/A	N/A	N/A	N/A	N/A	N/A
pAA-EA11	2050	1	0	1	0	0	0	0
pG-EA11	2050	0	0	0	0	0	0	0
3493_chromosome	2071	253	53	126	74	15	18	34318
pESBL-EA11	2071	N/A	N/A	N/A	N/A	N/A	N/A	N/A
pAAL-EA11	2071	3	2	1	0	0	0	0
pG-EA11	2071	0	0	0	0	0	0	0
2050_chromosome	2071	37	31	2	4	9	3	12496
p09EL50	2071	N/A	N/A	N/A	N/A	N/A	N/A	N/A
pAA-09EL50	2071	2	2	0	0	0	0	0
pG-09EL50	2071	0	0	0	0	0	0	0

### Pulse Field Gel Electrophoresis of Plasmid DNA

For each sample 20 µl of eluted plasmid DNA from 3 ml culture volume was analyzed by pulse field gel electrophoresis (PFGE). Briefly, 20 µl eluted sample was mixed with 4 ul of 6X loading dye (US Biologicals, Swampscott, MA) and loaded onto a 1% Pulse Field Certified Agarose (BioRad Laboratories, Hercules, CA) gel. Ten µl of high molecular range DNA ladder (5 kb DNA size standard, BioRad Laboratories, Hercules, CA) was loaded onto a lane as a size standard in the gel. The gel was electrophoresed in 0.5× TBE buffer, recirculated at 14°C. The run time was 18 hours at 6 V/cm with a 1 to 6 second switch time ramp. After completion of run the gel was stained with 500 ml of ethidium bromide (EtBr) solution (10 µg EtBr/ml of distilled water) for 1 hour at room temperature. The gel was de-stained with 1 liter of distilled water for 1 hour at room temperature prior to visualization in a UV transilluminator (BioRad) and photographed.

**Table 5:** List of loci disrupted, deleted, or replaced by IS elements.

		Strain (GenBank Locus Tag)			
Reference Locus Tagsor Coordinates	Function of reference genes	TY_2482	2011C–3493 (O3K_)	2009EL–2050 (O3M_)	2009EL–2071 (O3O_)
O3K_00120	Hypothetical transcriptionalregulator yidL			IS interrupted (O3M_00125–O3M_00130)	IS interrupted (O3O_25495–O3O_25490)
O3M_00415; O3M_00385–O3M_00455	hypothetical protein HMPREF9535_03951&Tet/Mer loci	IS interrupted	IS interrupted (O3K_00405)		Tet/Mer loci deletion (O3O_25240)
O3M_00970–O3M_00990	Ascorbate/lyxose-metabolism				Ascorbate/lyxose loci replaced by IS (O3O_24725–O3O_24720)
O3K_01350 & O3M_01375	Transcriptional activator GadE				IS interrupted (O3O_24325–O3O_24320)
O3K_01485	conserved hypothetical protein			IS interrupted (O3M_01515–O3M_01525)	IS interrupted (O3O_24175–O3O_24165)
O3K_07460 & O3M_07510	Putative HTH-type transcriptional regulator ypdC				IS interrupted (3769133.3770114)
2009EL-2050: O3M_11300; 2009EL–2071∶2336934.2336934	YdjO protein	IS interrupted	IS interrupted (O3K_11325)		
2011C–3493∶4620537.4618976	Protein YjgL, putative CCAAT-box DNA binding protein subunit B			IS interrupted (O3M_22275–O3M_22280)	IS interrupted (O3O_03015–O3O_03010)
O3K_07650–O3K_07740	Prophage elements			Prophage elements deletion by IS (O3M_07690)	
O3K_08905	Putative fimbrial-like adhesinprotein StcD			IS interrupted (O3M_08860–O3M_08865)	IS interrupted (O3O_16775 –O3O_16720)
O3K_11150	hypothetical protein			IS interrupted (O3M_11125)	IS interrupted (O3O_14470)
O3K_13005	putative sulfatase			IS interrupted (O3M_12965)	IS interrupted (O3O_12630)
O3K_19985	Predicted membrane protein			IS interrupted (O3M_19970)	IS interrupted (O3O_05310)
O3K_20190	hypothetical protein			IS interrupted (O3M_20085)	IS interrupted (O3O_05195)

### Whole-genome Sequencing

Genomic DNA was prepared using the Ultraclean Microbial DNA isolation kit (MoBio, Carlsbad CA). The draft genome sequences of all three isolates were generated by a consortium consisting of ECBC, NMRC and the LANL Genome Science Group using a combination of Illumina [40] and 454 technologies [41]. For each of these genomes we constructed and sequenced Illumina and 454 Titanium standard shotgun libraries, and a paired end 454 library (Table S1 and S2 in File S2). All general processes and protocols of library construction and sequencing can be found at http://www.jgi.doe.gov/. The 454 Titanium standard data and the 454 paired end data were assembled together with Newbler, version 2.3-PreRelease-6/30/2009. The Newbler consensus sequences were computationally shredded into 2 kb overlapping fake reads (shreds). Illumina sequencing data was assembled with VELVET, version 1.0.13 [42], and the consensus sequence were computationally shredded into 1.5 kb overlapping fake reads (shreds). The 454 Newbler consensus shreds, the Illumina VELVET consensus shreds and the read pairs in the 454 paired end library were integrated using parallel phrap, version 1.080812 (High Performance Software, LLC) [43], [44]. Illumina data was used to correct potential base errors and increase consensus quality using the software Polisher developed at JGI (Alla Lapidus, unpublished). Possible mis-assemblies were corrected using gapResolution (Cliff Han, unpublished), or Dupfinisher [45], and further edited manually with Consed [46]. The initial high quality draft assemblies contained 68–77 contigs and 6–9 scaffolds. Gaps between the contigs were closed by editing in Consed, by PCR, and by primer walks. Each genome required 400–800 additional finishing reactions to close gaps, resolve repetitive elements, and correct low-quality sequence regions. The final assemblies are based on 84.1–181.1 Mb of 454 draft data which provides an average 16.2–34.8×coverage of the genome and 1,590 Mb of Illumina draft data which provides an average 305.8× –1,246.4x coverage of the genome. The final genomes are of finished quality [47] whose structures were verified using paired-end read mapping.

**Figure 3:**
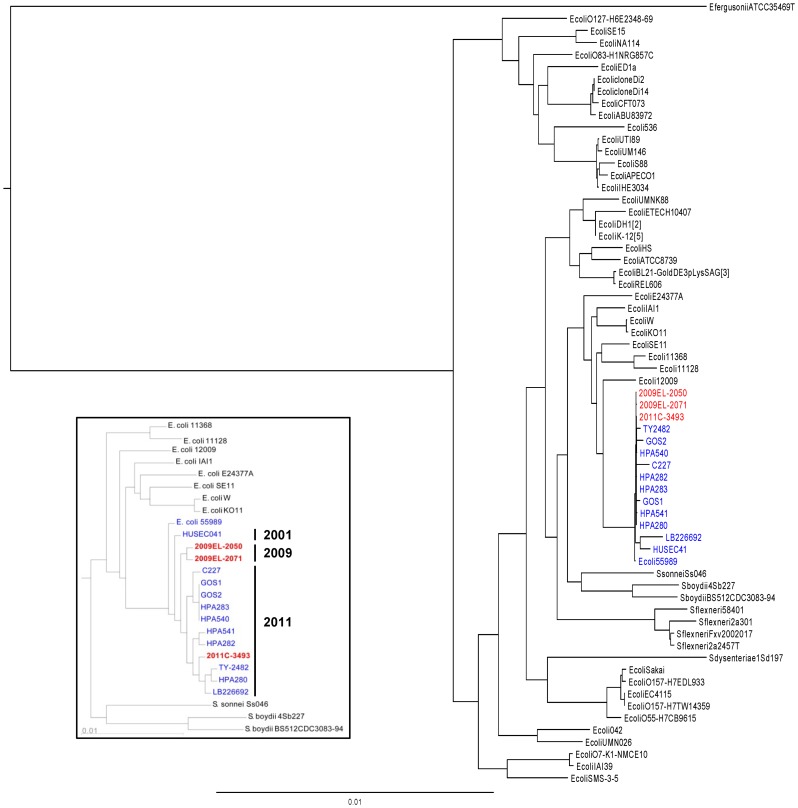
Georgian strains cluster with 2011 European outbreak strains. Phylogenetic comparisons were carried using all core *E. coli*Inset: Phylogenic analysis of subset of most closely related strains using all conserved orthologs. O104:H4 isolates are labeled in blue type; strains sequenced for this study are indicated in red type. Maximum likelihood phylogenies were constructed using RAxML (*E. coli-*wide tree) or Fasttree (inset) as described in Methods.

### Optical Mapping

Confirmatory optical maps were generated according to manufacturer’s procedures (OpGen, Gaithersburg MD) using *Nco*I and/or *Bam*HI. Optical maps of the strains were compared to each other and to the previously published TY-2482 sequence using MapSolver (OpGen).

### Identification of SNPs and Small-scale Genetic Variations

The finished sequences were compared to each other using the nucmer algorithm in MUMMER [48], [49]. SNPs and Indels were reported directly from genome alignments while the unaligned portions of the respective genomes were tracked by coordinates and captured as gaps. Candidate variations in the nucleotide sequences in the finished sequences of each strain were confirmed by mapping the raw 454 and Illumina reads back onto the finished sequence using the GSMapper package in Newbler and/or the read-mapping tool in Genomics Workbench from CLC Bio. Variations evident in both the finished and mapped data and free of potential assembly conflicts (i.e. where >85% of the raw reads differed from the reference) were considered to be confirmed. Instances where the raw sequencing data from each strain conflicted with the finished sequence from the parent strain were considered to be errors in the final assembly.

**Figure 4:**
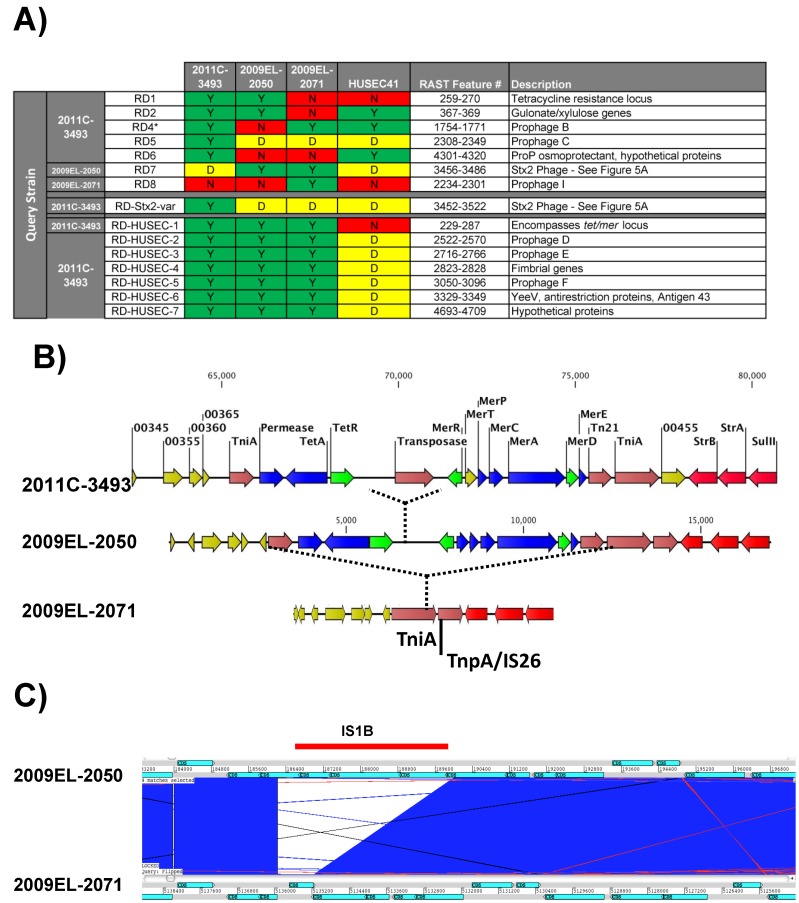
A unique repertoire of genomic islands is present in each strain. A) Identification of chromosomal Regions of Divergence for each strain. Pair-wise BLASTp analysis of annotated protein sequences in RAST was used to determine reciprocal best-hits in each strain. Each closed, complete strain was used as a query against the other strains. RDs were defined as regions exhibiting 4 or more adjacent genes that were absent (red) or significantly divergent (<99% identical at the protein level) (yellow) in the target strains. ^*^Annotation differences initially showed an RD3 locus but subsequent BLASTn alignments did not confirm. **B)** The Tet/Mer locus of the 2011 and 2009 outbreak strains. Genes encoding resistance are shown in red, transport/efflux functions are indicated in blue, regulatory functions in green, and transposon functions in dark red. Hypothetical proteins and proteins of unknown function are indicated in yellow. **C)** Loss of ascorbate/lyxose-metabolism genes from 2009EL-2071 due to insertion of an IS1B element.

### Phylogenetic Analysis

The MCL clustering algorithm [50] was used to identify conserved core protein ortholog families between the German outbreak isolate 2011C–3493, TY-2482 strain, the two Georgian isolates 2009EL–2050, and 2009EL–2071 and all complete *E. coli* genomes available in NCBI (Table S3 in File S2). *Escherichia fergusonii ATCC 35469T* strain was used as an outgroup. Ortholog families with genes present in all *E. coli* as well as the German and Georgian isolates were used to create the core phylogeny. Protein sequences in each family were aligned separately using MAFFT [51], then concatenated by species into a mega-alignments using an in-house perl script and removing uninformative columns. A phylogenetic tree was constructed using RAxML [52] with the JTT+GAMMA+I model. This same approach was applied to the O104:H4 clade to highlight relationships within this group of closely related organisms using FasttreeMP and the general time-reversible model [53].

**Table 6:** Location and identity of prophages in the *E. coli* genome sequences characterized in this study.

			Strain											
			2011C–3493			TY2482			2009EL–2050			2009EL–2071		
Pro-phage	*att* site	# ORFs	Start^1^	Stop^1^	Length	Start	Stop	Length	Start	Stop	Length	Start	Stop	Length
**A**	N.D.	36	950325	909830*	40496	*953117*	*912171*	40947	*953772*	*912076*	41697	*943452*	*901106*	42347
**B**	tRNA-Arg	35	1604666	1577150	27517	*1607949*	*1580433*	27517	*1607949*	*1580433*	27517	*1599640*	*1572124*	27517
**C**	N.D	65	2148635*	2195980	47346	*2199458*	*2152036*	47423	N/A^2^	N/A	N/A	N/A	N/A	N/A
**D**	N.D.	52	2393440*	2355821	37620	*2396966*	*2359347*	37620	*2382874*	*2346454*	36421	*2390590*	*2354170*	36421
**E**	*rpsB/dmsB*	81	2595666	2525555	70112	*2599378*	*2529267*	70112	*2585195*	*2515084*	70112	*2592911*	*2522800*	70112
**F**	N.D.	69	2939977*	2886708	53270	*2943775*	*2890454*	53322	*2931484*	*2878212*	53273	*2938762*	*2885152*	53611
**G (** ***stx2A*** **)**	*wrbA*	85	3317120	3248372	68749	*3321914*	*3253022*	68893	*3309116*	*3240628*	68489	*3366761*	*3298273*	68489
**H**	*ybhC*	61	3581902*	3624519	42618	*3586627*	*3629642*	43016	*3576100*	*3616522*	40423	*3633744*	*3674166*	40423
**I** 2	tRNA-Arg	70	N/A	N/A		N/A	N/A		2187036	2137953	49084	2194752	2145663	49090
**J**	*potB/potC*	71	N/A	N/A		N/A	N/A		N/A	N/A		3102419	3052917	49503

1 Start/Stop coordinates are normalized to the first C nucleotide of the sequence CATTATCGACTTTTGTTCGAGTGGAGTCC.

2 Prohage I has significant homology to prophage C.

* PhageFinder start/stop output was manually curated by inspection of RAST-annotated chromosomes.

*Italics* indicate identification of phage start/stop sites by BLAST using 2011C–3493 manually curated PhageFinder output as query.

### Genomic Comparison

The genome comparisons at the nucleotide level were carried out with genome alignment tools, such as MUMmer2 [48], NUCmer [49], and the Artemis Comparison Tool (ACT) [54] (http://www.sanger.ac.uk/Software/ACT/). The comparison of genomic island insertion/deletion patterns was identified using the ACT alignment program at the default settings. Predicted genomic island insertion sites were identified from sequence alignments and breakpoint sites were further manually curated. The gene name and locus ID were assigned based on the NCBI Reference Sequence file.

**Figure 5:**
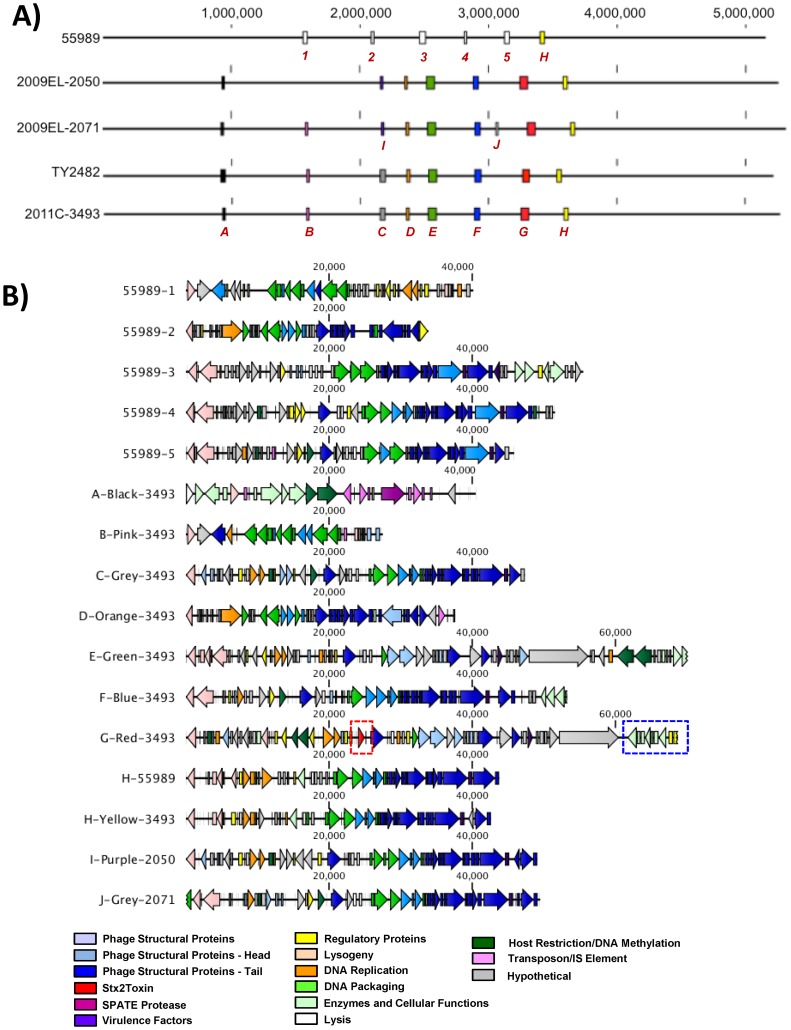
Analysis of prophage content of O104:H4 strains. **A)** Location of prophages in the genomes of the EAggEc strains analyzed in this study. Linear maps of the genomes and the location of prophages as boxes are shown. All genome sequences have the same starting position as described in methods. The prophage locations are drawn to scale. Phages are color-coded according to similarity; for example the red box indicates the stx2a phages. The exact genomic locations of the prophages in their respective genomes are given in Table 6. **B)** Architecture of individual prophages. Phage proteins are colored according to their predicted functions. The *stx2ab* genes are boxed in red; the island of pyrimidine biosynthesis genes identified as a part of this prophage by Phage_Finder is indicated by the blue box. In all cases the *int* genes are positioned on the left, regardless of the orientation of the prophage within the chromosome.

**Table 7:** Comparison of putative prophages to NCBI database using BLASTn.

	NCBI Accession	QueryCoverage(%)	% Identity1	LongestHit (nt)	Description
**A**	N/A				Contains mainly host-derived genes, mobile elements with a single integrase homolog. Probable False-positive [56]
**B**	AF547987.1	51	97	10552	Enterobacteria phage Sf6, complete genome
**C**	M81255.1	16	96	6464	Bacteriophage 21 head gene operon^2^
**D**	AF034975.3	4	86	1677	Bacteriophage H-19B essential recombination function (erf), kil protein, regulator
**E**	AB255436.1	49	93	12504	Stx2-converting phage 86 DNA, complete genome
**F**	M81255.1	14	95	7587	Bacteriophage 21 head gene operon^2^
**G (** ***stx2A*** **)**	JQ011318.1	81	99	22430	Escherichia phage TL-2011c, complete genome [31]
**H**	EU078592.1	61	97	18369	Enterobacteria phage DE3, complete genome
**I^‡^**	EU078592.1	22	98	10194	Enterobacteria phage DE3, complete genome
**J**	M81255.1	14	94	6337	Bacteriophage 21 head gene operon^3^

1 % identity of longest BLAST hit.

2 Also hit to EU078592.1 (Enterobacteria phage DE3) and J02459.1 (Enterobacteria phage lambda).

3 Also hits to AJ556162.1 (Phage BP-4795) and FM180578.1 (Enterobacteria phage 2851).

### Identification of Prophage Regions in Completed Genome Sequences

The finished genomic sequences of the three strains focused on in this study along with the published sequences of 55989 (NC_011748.1) and TY2482 (PRJNA67657) were annotated using the DIYA software [55]. The GenBank outputs of DIYA software were used as inputs for Phage_Finder analysis software [56] to identify potential prophage regions in the genomic sequences. The Phage_Finder outputs were manually curated to identify additional phage proteins outside of the regions identified by Phage_Finder and to validate the identity of the phage related genes encoded by the prophages [57]. Prophage similarity analysis was conducted using BLASTN version 2.2.18 [58]. Phage similarity was defined as any two phages having 95% or greater identity along 95% or more of genome length and position in the same general location on the *E. coli* chromosome. The manually curated Phage_Finder outputs sequences were further validated by BLASTn analysis of the whole-genome sequences of all five strains to establish uniqueness of each prophage and also to account for potential prophage sequences that might have been missed by Phage finder. To generate this image all the sequences were rearranged to start at the same nucleotide position. Accordingly, the genome position 1 was set at the first C nucleotide in the following sequence CATTATCGACTTTTGTTCGAGTGGAGTCC. Extracted phage sequences were aligned to each other using the multiple alignment tool in CLC Bio.

**Figure 6:**
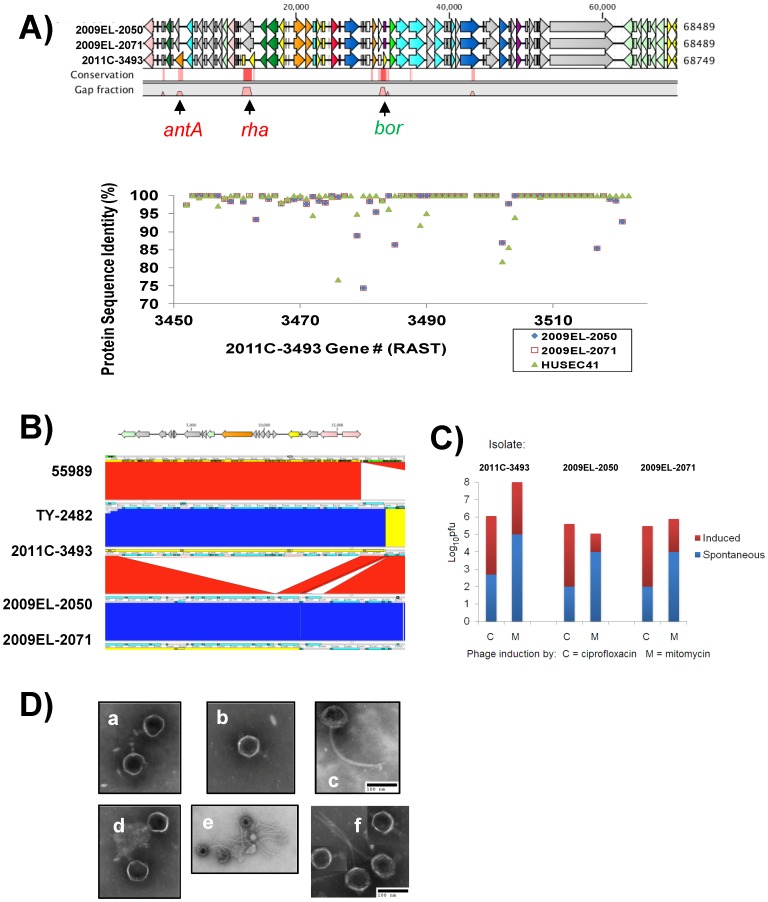
Genetic divergence of phage loci and induction of phage particles. A) Differences in Shiga toxin phage genome organization and sequence. *Top panel* - Comparison of stx prophage region. stx2 prophage genomes were aligned in CLC Bio using the multiple alignment tool. Gaps in the regions of homology are indicated by pink spikes while areas of sequence divergence (including gaps) are indicated in red hashes. Red and green lettering indicate genes that are present in the Georgian strains but not in the European outbreak strain and *vice versa*, respectively. *Bottom panel* – Pairwise comparison of Stx2 phage protein orthologs in RAST relative to strain 2011C–3493. **B)** Deletion of small prophage region from Georgian isolates. **C)** Induction of infectious phage particles from 2011 and 2009 strains. **D)** Heterogeneous phage morphotypes are evident upon induction of phage from (a) 2011C–3493, induced with mitomycin C (b) 2009EL–2050, spontaneous (c) 2009EL–2071, induced with ciprofloxacin, (d) 2009EL–2071, induced with mitomycin C, (e) 2009EL–2050, induced with ciprofloxacin, (f) 2009EL–2071, induced with ciprofloxacin.

### Phenotypic Analysis

Each strain was inoculated into twenty 96-well OmniLog phenotypic microarray plates and grown at 37°C for 36 hours. Reduction of tetrazolium dye by respiring cells was measured every 15 minutes by optical density. A heatmap of the data was produced using PheMaDB [59]. Briefly, the area-under-the-curve (AUC) values from the three different biological replicates for each unique phenotype were averaged. The ratio for each AUC was calculated between the query strain and reference parent strain. For the purpose of visualization, 1920 phenotypes were included in the heat map (i.e. this better represents the locations of the phenotypes which correspond to different modes of action categories). The same ratios were used for the phenotypes that have replicates. The ratio values were formatted as PM1 to PM20 for each strain across the rows and wells A*_i_* to H*_i_*, where *i* = 1 to 12 for the columns (note that there were no values for wells H12). The results were plotted in a heat map using R [60]. Wells in which the query strain outgrew the reference strain are represented by green blocks while wells in which the reference strain outgrew the query strain are represented by red blocks.

**Figure 7:**
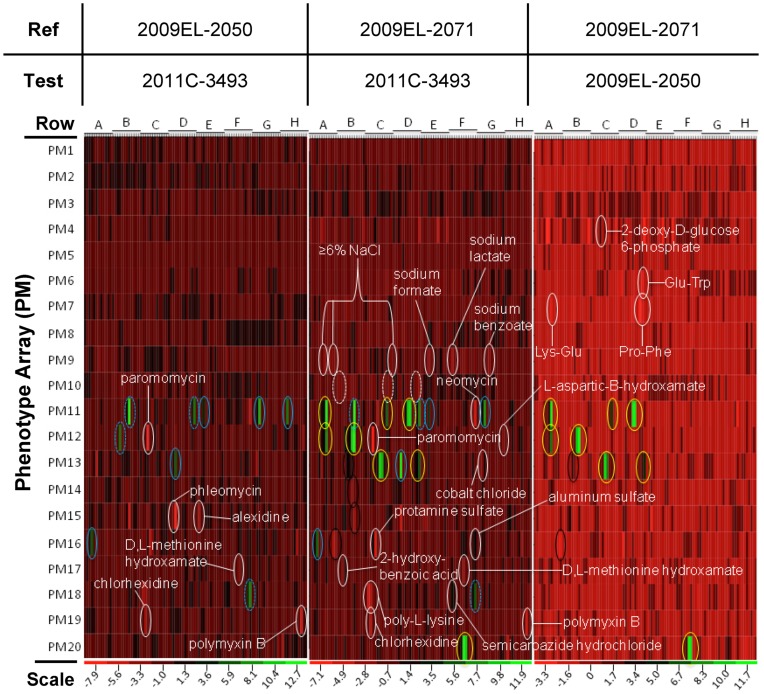
Pair-wise heat map phenotypic comparison of three *E.coli* strains. Each strain was assayed for growth in the presence of various chemicals using OmniLog phenotypic microarrays, as detailed in Materials and Methods. Each well represents the average of three biological replicates. The columns represent the well position, and are denoted as A_i_ to H_i_ (i = 1 to 12) from the left to the right of the plot in each array along the x-axis (note that there were no values for wells H12). Each cell ratio value represents the average of three biological replicates. Plates PM01–PM10 contains single wells for each growth condition, while plates PM11–PM20 contain quadruplicate wells for each growth condition. Those wells which exhibited a two-fold or greater difference in growth and which were statistically significant, with P value less than 0.05 (See File S5), are indicated here. White dashed ovals indicate pH of 4.5, tetracyclines are indicated by a yellow oval, cephalosporins by a solid blue oval, lactams by a dashed blue oval, and chelators by a black oval. The apparent variation in the heat map in the comparison of 2009EL–2050 and 2009EL–2071 is a function of the reduced scale of the heat-map (−3.3 minimum value).

### Phage Induction

Bacterial strains were streaked for single colonies on tryptic soy agar plates from −70°C stocks and were incubated at 37°C for 18 hours. After initial incubation, single large isolated colonies were inoculated into the following media: 20 ml tryptic soy broth containing 25 µg/ml of mitomycin C, 4 µg/ml ciprofloxacin, or no antibiotics. Liquid cultures were grown at 37°C on a shaker for 18 hours. After overnight growth, 2 ml aliquots from each culture were taken and centrifuged at 10,000×g for 2 minutes to pellet the bacteria. Supernatants were then collected and transferred to 0.22 µm Spin-X centrifuge filter tubes and centrifuged at 10,000×g for another 2 minutes to filter sterilize. When not in use, supernatants were stored at 4°C. To estimate the number of viable phage particles produced spontaneously or under inducing conditions, supernatants were titered on the susceptible naïve indicator *E. coli* strain DH5α. For titration, *E. coli* DH5α was inoculated into 10 ml tryptic soy broth and grown at 37°C to an optical density (OD_600_) of ∼ 0.5. 100 µl of the DH5α culture was infected with 10 µl of 10-fold serial dilutions of the supernatant from uninduced and induced cultures and incubated at 37°C for 20 minutes for phage adsorption. 2.5 ml of molten top agar kept at 48°C was added to the bacterial-phage mix and immediately poured onto tryptic soy agar plates pre-warmed to 37°C. The top agar was allowed to solidify for 5 minutes at room temperature before incubating for 18 hours at 37°C. Plates were examined after overnight incubation and plaques were enumerated.

**Figure 8:**
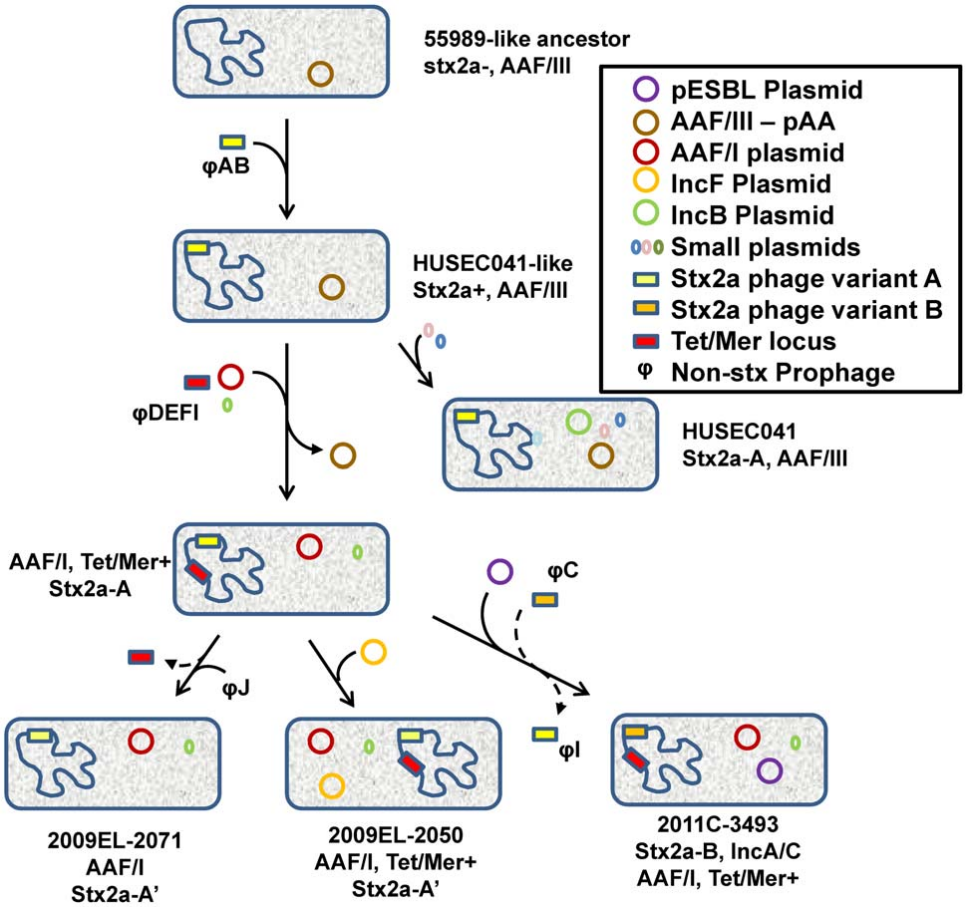
Model for evolution of the 2009 and 2011 *E. coli* O104:H4 strains. An ancestor enteroaggregtaive strain similar to 55989 acquires a Stx2a phage (Stx2a-A), then an IncB plasmid to become the HUSEC041 strain, diverging from the lineage of strains which led to the European and Georgian strains. Those strains acquire the *tet/mer* resistance locus, replace the AAF/III plasmid with the AAF/I plasmid observed in all three strains, and acquire a cryptic 1.5 kb plasmid. 2009EL–2071 loses the *tet/mer* locus. 2009EL-2050 also acquires an IncF plasmid. 2011C–3493 acquires both an ESBL-expressing plasmid, and the Stx2a phage that initially infected the strain is displaced by a second Stx2a phage (Stx2a-B).

## Results

### Traditional Tests and PFGE

Selected results of a panel of biochemical and phenotypic tests are given in Table 2, with results from the complete panel of tests in File S1. Most phenotypes of the three strains were similar.

### Antibiotic Resistance

Antibiotic resistance profiles were determined for all three strains (Table 2). The 2011 outbreak isolate was shown to be resistant to several antibiotics in the cephalosporin class. As in previous reports, these resistance elements correlated with the presence of a CTX-M-15 cephalosporinase encoded on the IncI1 plasmid that was present only in the 2011 outbreak strain. In addition, two of the strains were resistant to tetracycline, consistent with the presence of a *mer/tet* cluster in both strains. Multiphenotypic analysis using Omnilog (see below) confirmed this result, but also indicated that the 2011 strain is more sensitive to a number of membrane-disrupting agents including polymyxin B, chlorhexidine and paramomycin.

### PFGE Analysis of Plasmid DNA

PFGE analysis of extracted plasmid DNA revealed that the 2011 outbreak strain (2011C-3493) contains 3 different sized plasmids (Figure 1A). The large plasmid is approximately 90kbp, the intermediate plasmid is ∼75 kbp and the small one is approximately ∼1.5 kbp. These three plasmids were reported to be present in the 2011 outbreak strains characterized in other studies [36], [37], [38]. The two Georgian strains, 2009EL–2050 and 2009EL–2071, lacked the ∼90 kbp plasmid but carried the 75 kbp and 1.5 kbp plasmids. Interestingly, strain 2009EL–2050 carried a ∼110 kbp plasmid. This plasmid, designated p09EL50, is absent from 2011C–3493 and 2009–EL2071 (Table S4 in File S2). The PFGE results are fully supported by whole genome sequence data described below.

### Whole Genome Sequencing

Summary genome sequencing statistics for each replicon and additional statistics describing the Illumina and 454 paired-end datasets each strain are presented in Table S4 in File S2. We compared our finished sequence of the 2011 strain to the previously released TY2482 genome sequence and, where applicable, to other published sequences.

### Plasmids

Plasmid sequences were compared using BLASTn to the nucleotide database at NCBI to identify their closest matches, which are given in Table 3. The plasmids and chromosome of Strain 2011C–3493 match closely to the previously published sequence of the TY2482 strain [38]. All three strains contained the small 1549 bp plasmid previously reported as pG2011 TY2482 [38] and referred to here as pG [61]. In addition, all three strains contained a ∼72 kb plasmid homologous to pAA TY2482 [6], which we refer to as pAA [61]. The pAA variant in 2009EL–2071 (pAA–09EL71) contained an additional insertion caused by the expansion of a 944 bp tandem repeat that is also present on the chromosome of a transposon/retron element (Figure 1B). The plasmid originally referred to as pESBL TY2482 [38] and referred to here as pESBL-EA11 was found only in the 2011 outbreak isolate 2011C–3494 and encodes the CTX-M-15 cephalosporinase. Strain 2009EL–2050 contained an additional IncF plasmid (p09EL50) that exhibited similarity to plasmids pLF82 and pHCM2 from the Adherent/Invasive *E. coli* strain LF82 [62] and *Salmonella enterica* subsp. e*nterica* serovar Typhi, respectively [63] (Figure 1C). The plasmid component of the Georgian strains was yet further distinct from the recently sequenced HUSEC041 strain, another O104:H4 StxEAggEC strain from a 2001 case in Germany [64]. Interestingly, the 1.5 kb pG TY2482 plasmid varied dramatically in copy number when coverage of each replicon in the datasets was compared (Table S4 in File S2). The read coverage of this replicon was not consistent with the quantity of plasmid recovered from this isolate (Figure 1A), suggesting that differences in growth conditions may affect the apparent plasmid copy number.

### Chromosome

To probe the relatedness of these isolates at the chromosomal level, we examined the chromosomal architecture of the three strains. To provide verification of the accuracy of our assemblies, large-scale assemblies were verified by comparison of the finished genomes to in-house generated optical maps for all three strains which matched the finished sequences for all three isolates (File S3). The chromosomes are very similar in overall architecture (Figure 2A) with no gross rearrangements detected when the chromosomes were aligned using MAUVE. A comparison between the chromosomes of TY2482 and 2011C–3493 showed only 29 SNPs (14 synonymous, 9 non-synonymous, and 6 intergenic) and 7 gaps (totaling 859 bp), with an additional 16 SNPs (all in intergenic regions) and 3 gaps (totaling 104 bp) identified between their plasmids pESBL TY2482 and pESBL-EA11 (Figure 2B, File S4). Most of the SNPs between TY2482 and 2011C–3493 were clustered in a putative prophage region which may indicate a misassembly in the TY2482 genome sequence or rapid divergence of this region. All but two of the remaining SNPs (at positions 43333 and 1568661 (TY2482 coordinates) were identified previously as sequencing errors in the TY2482 reference [65]. Notably, 2011C–3493 shared the sequence with TY2482 at position 2252380 in the L-asparaginase 2 gene that delineated the German strains from other European isolates [65].

All gaps, small indels, and SNPs (including information on intergenic, synonymous or non-synonymous SNPs) found in all two-way comparisons between the finished German and Georgian genomes are enumerated in Table 4. The intersection of these differences show lineage specific differences between the strains. A comparison between chromosomal sequences of 2011C–3493 isolate and 2009EL–2050 show a total of 262 SNPs (61 present in the intergenic regions, 127 synonymous, and 74 non-synonymous), and 253 SNPs (53 present in the intergenic regions, 126 synonymous, and 74 non-synonymous) present between 2011C–3493 and 2009EL–2071 isolates (File S4). Most of these SNPs (245) were shared between the two Georgian strains (Figure 1B), with only 7 and 16 SNPs unique to 2009EL–2050 and 2009EL–2071, respectively. When 2009EL–2050 and 2009EL–2071 are compared to each other, the strains are differentiated by 37 SNPs (31 intergenic, 2 synonymous, 4 non-synonymous), 31 of which cluster around a multi-tRNA locus. In addition, 3 gaps (12kbp total) were identified between the Georgian strains. These gaps represent genomic islands and deletions (see below) that differentiate the strains, and confirmed results using optical maps (ref. [66] and File S3). Many of the SNPs between the two Georgian strains and between each of the Georgian strains and 2011C-3493 cluster around putative prophage elements (see below), and are indicative of the divergent temperate phage residing in the chromosomes of these strains. However, other SNPs occur in core regions of the genome, providing evidence of divergence of the strains from a recent common ancestor.

### Disrupted Genes

The 2011 and 2009 strains each contained a unique set of insertion elements at different positions on the chromosome (Figure 2D). The 2011 strain had a unique profile of IS element insertions, while both Georgian strains appeared more similar to each other. A number of unique insertions resulted in disruption of genes due to mobilization of elements such as transposons (Figure 2D; Table 5). Most of these insertions occurred in genes of unknown function, but several of the interrupted genes have homology to enzymes or transcriptional regulators (Table 5). Finally, several IS elements present in the plasmids of the Georgian strains are missing from the plasmid component of the 2011 outbreak isolate. Using IS element proliferation as a surrogate for genomic decay, this suggests that the plasmid present in the 2011 outbreak strain may have suffered less genomic decay than the Georgian strains. In addition to gene loss incurred by IS element transposition, each strain contains a unique repertoire of pseudogenes arising by nonsense or frameshift mutations (File S4). As with the IS elements, the profiles of these disrupted genes in the Georgian strains are much more similar to each other than they are to the 2011 outbreak strains. 2009EL–2071 in particular contains an interrupted *gadE* gene that is truncated by the insertion of an IS element. The transcriptional activator GadE regulates the transcription of genes involved in the maintenance of pH homeostasis during acid stress by controlling the decarboxylation of glutamine [67], [68].

### Phylogenetic Analysis

The pan-genome of all complete genomes of *E. coli* available in GenBank along with the German 2011 (2011C–3494) and Georgian 2009 (2009EL–2050, 2009EL–2071) outbreak isolates consisted of 16,806 protein families. Of these, 1,136 families are shared among all strains and considered as the core genome of *E. coli*. The protein sequences in each family were aligned separately and then concatenated by species. A phylogenetic tree was inferred from this core and as expected, the two Georgian isolates clustered closely with the 2011 outbreak strains (Figure 3). To allow better differentiation of the Georgian from the 2011 outbreak isolates, we used a similar approach to find all conserved orthologs from the O104:H4 clade (2323 protein families) and used the concatenated O104:H4 core sequences to obtain a phylogenetic tree. The Georgian strains formed a cluster distinct from the 2011 outbreak isolates (Figure 3, inset), while 2011C–3493 clustered with the other extant sequences from various European outbreak strains.

### Genomic Islands

Several genomic islands and large genetic elements were found that differentiated the Georgian from the 2011 outbreak strains (Figure 2A, arrowheads). In addition to the variation in plasmid content, several large regions of divergence (RDs) were observed between the strains (Figure 4A). These included prophages and large insertions/deletions mediated by recombination events and/or mobile genetic elements. These are described in more detail below. Several additional islands were noted that were common to all of the strains examined in this study but absent from the draft sequence of the 2001 HUSEC041 isolate [29].

- *Mercuric ion/tetracycline resistance island (RD1) –* Both the 2011 (2011C–3494) and one of the 2009 Georgian isolates (2009EL–2050) contained an intact mercuric ion resistance locus. The 2011 strain contains an additional insertion of an IS element that separates the *tet* and *mer* operons. In the second Georgian isolate, this region appears to have been deleted by recombination between terminal repeat regions (Figure 4B). In general, the gene content of the RD1 locus corresponds to the detection of those genes in the Georgian strains by microarray and PCR previously reported by Jackson and co-workers [66].
- *Ula operon (RD2) -* One potentially significant loss of activity in 2009EL–2071 is in an operon containing homologs of genes involved in anaerobic degradation of ascorbate [69] (Figure 4C). We did not observe a phenotype for growth on ascorbate; however this is likely due to the use of aerobic growth conditions in our phenotype array experiments.

### Integrated Prophages

To determine the nature and identity of potential prophages in our genome sequences, we utilized Phage_Finder, an automated bioinformatic algorithm designed to identify potential integrated prophage sequences [56]. Phage_Finder identifies regions of homology to a curated database of protein sequences and functional domains commonly associated with bacteriophage. The 2011 European outbreak strains, the Georgian strains, and strain 55989 were interrogated using Phage_Finder, which identified a total of 38 regions in all five genome sequences that could encode putative prophage regions (Table 6). Phage_Finder identified a total of 38 phage-like regions in the genomes queried and assigned putative left and right termini. However, to compensate for inherent biases in the algorithm, which had previously caused Phage_Finder to over- or under-call the number of potential prohage-encoded ORFs in 50% of genomes in its training dataset [56], the outputs were curated manually to identify regions outside of the termini identified by Phage_Finder. Manual inspection revealed several additional regions that had been annotated by RAST as potential genes encoding phage-related functions. Most of these genes missed by Phage_Finder encoded structural functions such as head and tail proteins, but in a few cases lysogeny and integrase functions were missed (not shown). Phage_Finder also identified potential attachment (*att*) sites of several of the prophage sequences (for details see Table S5 in File S2). Some of these *att* sites were utilized by different phages in different strains; for example prophage 55989-1 appears to occupy the same site as prophage B in the *stx2a-*positive strains. One potential false-positive region was identified, notated as Phage A in Figure 5, which contains few phage related genes (only a single integrase homolog was found); however several mobile elements and a putative restriction-modification system were identified within this region which may account for its being identified by Phage_Finder. Interestingly, prophage region A encodes one of the four SPATE protein homologs present in these strains, suggesting possible transfer of this element into these strains via a mobile element.

Once identified in a single strain, phage sequences were utilized as BLASTn queries against the other finished genomes (including TY2482) and against the NCBI database, limiting the database to double-stranded DNA viruses with no RNA intermediate (NCBI Taxonomy ID #35237). The results of the BLAST analyses are shown in Table 7. For queries against the other bacterial strains, we set a cutoff of 95% identity over 95% of the length of the prophage genome for assigning the putative prophage as the same phage. A total of 15 discrete prophages were identified, only one of which (phage H) was common to all five strains (Figure 5A). The *stx2*a-positive strains had six phages in common (A, D, E, F, G, H), with prophage B missing from 2009EL–2050. The two Georgian strains had replaced prophage C with prophage I; these two phages share extensive homology and may be distantly related variants or mosaics with another phage (File S5). One additional prophage sequence (prophage J) was observed in 2009EL–2071. There are some minor discrepancies in the sizes of the prophages between 2011C–3493 and the previously reported TY2482 sequence. These are likely due to minor misassemblies in the TY2482 genome sequence, which is a draft assembly in contrast to that of 2011C-3493, which is a finished sequence.

Most of the other prophage regions identified by phage-finder contain a sizable repertoire of structural and non-structural phage genes, and exhibit a variety of architectures, mostly lambdoid in nature (Figure 5B). Two of the putative prophage (E and G), exhibit architectures similar to typical Stx-converting phage [70]; indeed phage G encodes the *stx2* genes. These prophages contain a distinctive gene that encodes a long protein (∼2800 amino acids) of unknown function that is common to other stx2 phages [70]. In addition to the Shiga toxin genes themselves, several of the prophage encode potential virulence factors that may be present as phage morons (proteins encoded by phage that play no role in phage replication or structure yet confer upon the host bacterium important evolutionary advantages, such as during virulence). These include homologs of the *ail/lom* gene family (Phages C, F, G, H, I, J), and the *bor* (Phage G) genes. The *ail/lom/bor* genes belong to a family of enterobacteral outer membrane proteins expressed by lambda-like phage that confer eukaryotic cell invasion [71] and/or resistance to serum-mediated killing [72], [73].

### Structural Variation in Phage Regions Including the Shiga-toxin Phage

To determine the degree of divergence of the individual phage sequences within the finished genomes, we extracted the prophage sequences from the whole-genome sequence and aligned the sequences of each prophage individually (File S5). The results of this analysis revealed subtle structural variation in several of the prophages, most notably in the stx2 phage (Figure 6A). Two other phages showed more subtle variations (D, F, and H), which can be ascribed largely to the mobilization of an IS element (D) or to the presence of small deletions (F and H). Phages C and I, although highly similar, were considerably more divergent and suggested a chimeric structure. The effect on the protein coding sequences of the variations were not particularly dramatic for all but the Shiga-toxin phage (Figure 6A); notably the 2011C–3493 isolates contain a deletion that spans a portion of a *bor* homolog, which may be involved in serum complement resistance [72]. Other functions that may be perturbed in the Georgian isolates are an antirepressor protein homolog (*antA*) and a *rha* homolog, both of which are disrupted by deletions. Pairwise comparison of the protein sequences encoded by the stx2 phage (Figure 6A, bottom panel; see also File S6) revealed additional effects on protein sequence between 2011C–3493 and the two Georgian strains. The stx2 phages of the Georgian strains were otherwise indistinguishable from each other but for a single synonymous mutation in the *stx2A* gene.

A small region containing prophage-like genes in 2011C–3493 (positions 4128768–4145397 according to the normalized coordinate system in Fig. 5 and Table 6) is also present in strain 55989 and TY2482, but is absent from the Georgian strains (Figure 6B). Curiously this region was missed by Phage_Finder, in spite of the presence of genes encoding putative primase, integrase, and antitermination functions. Phage_Finder misses approximately 10% of known phage sequences, so this result is not surprising [56]. The lack of genes encoding obvious structural proteins suggests that this prophage region might be degenerate.

### Phage Induction

Given the extensive repertoire of prophages present in the *E. coli* outbreak strains, we asked if the prophages are cryptic or active and whether the strains produce viable phage particles that could explain the horizontal acquisition of stx2a phages by an EAggEc strain. It is well known that prophages, including the Shiga toxin-encoding stx2a phage, could be induced by growing the lysogenic strains in the presence of ciprofloxacin, mitomycin C, or by other stimuli [74]. Phage particles were isolated from uninduced and induced cultures and plated on an indicator *E. coli* strain DH5α. We observed an increase of several orders of magnitude of phage production upon induction with ciprofloxacin and mitomycin C (Figure 6C). The effect of inducing agents is much more pronounced in the case of mitomycin C and isolate 2011C–3493 compared to the 2009 isolates. The culture supernatants were examined by electron microscopy (Figure 6D) for the presence of phage particles. At least two distinct lambdoid phage morphologies could be observed. Both phage morphotypes exhibited an icosahedral capsid. One morphotype exhibited short tails (933W-like) (Figure 6D, panels a and b), while a second morphotype exhibited a typical *Siphoviridae*-like morphology with long, non-contractile tails (Figure 6D, panels c,e,f). The 933W-like morphotype was common among the 2009 and 2011 isolates. All of these morphotypes have been observed for prophages including stx phages induced from STEC strains [74]. The genomic analysis coupled with the isolation of distinct phage morphotypes indicate that multiple distinct, viable prophages are encoded within the genomes of these strains. Despite repeated attempts, we have thus far been unable to obtain stable stx2 phage lysogens of *E. coli* K-12 from the 2011 strain; we therefore cannot definitively assign a morphotype to the stx2 phage.

### High-throughput Phenotypic Analysis

In order to understand the functional differences between the three O104:H4 isolates, a high throughput phenotypic characterization was undertaken. We employed OmniLog Phenotypic Microarrays (PMs) and conducted a pair-wise comparison of the strains using the area under the curve (AUC) values that result from measuring the reduction of tetrazolium dye (as an indicator of growth) under the various conditions tested. AUC ratios and P values for each well for each pair-wise comparison were calculated and those that demonstrated a two-fold or greater increase or decrease in growth as compared to the parent strain and which were found to be statistically significant are presented in File S7, along with the chemical name and mode of action. Those wells that exhibited significantly different phenotypes are circled on a heat map display of the overall results in Figure 7. Along the bottom of each heat map panel is displayed a color coding scheme based on the range of values computed for the AUC ratio of test strain versus parent strain, and the color that represents each value per panel varies with range.

From this analysis, several trends were observed. Overall, the two Georgian isolates were found to be more similar to each other phenotypically than to the 2011 isolate, as evidenced by the limited range of the AUC ratios for this pair-wise comparison (−3.3 to 11.7) versus the other two comparisons (which had ranges of −7.9 to 12.7 and −7.1 to 11.9). Of the differences that were found amongst the three isolates, most differences were found in PM11–20, which assay for growth in presence of various antimicrobial compounds. These results were consistent with the genomic data. Isolate 2011C–3493 was found to be more resistant to cephalosporin (encircled by blue solid ovals) and beta-lactam (blue dashed ovals) antibiotics than either of the two Georgian isolates, probably due to the presence of the large IncI1 plasmid that encodes the CTX-M-15 cephalosporinase. Additionally, the Georgian isolate 2009EL–2071 is more sensitive to tetracyclines (yellow ovals) and less sensitive to chelating agents (black ovals) than the other 2 strains, consistent with the deletion of the *mer/tet* locus.

## Discussion

The severity of the 2011 outbreak centered in northern Germany and the high rate of progression to HUS among infected patients indicated that the O104:H4/HUSEC041 clade of StxEAggEC strains may represent a significant new threat to public health. As a function of ongoing biological and public health engagement and biosurveillance by the Georgian Centers for Disease Control and Public Health and their partner agencies in the United States, the analysis of several previously uncharacterized O104:H4 strains from a relatively unheralded cluster of cases in 2009 was undertaken. The analysis presented here reveals previously unreported genetic diversity among StxEAggEC strains and suggests that multiple lineages of such strains may currently be circulating worldwide. While this diversity was previously suggested by comparisons of gene content and optical maps between the 2011 and 2009 outbreak strains [66], the whole-genome sequences of these strains provide a high level of resolution and unambiguous placement for these genetic acquisitions and losses. Our results concur with those presented in a recent description of virulence factors present in a Georgian O104:H4 *stx2*-positive strain [75]. The strains of the European outbreak were shown to be clonal in a recent genomic epidemiology study [65]. While this isolate unquestionably belongs within the clonal group that includes the German isolates, the presence of previously unreported SNPs relative to the TY2482 genome in 2011C–3493 suggests strongly that at least some previously unsampled diversity is present within the German isolate group, although it is not clear at this time whether these two mutations were present in the population that seeded the outbreak or whether these represent the products of in-host evolution.

Considerable diversity is observed in the prophage component of these strains. This is not surprising, given the highly mobile nature of phage genomes and the prominent roles of phage in transferring genetic material between bacterial strains [76]. These differences are particularly evident in the related C and I prophages and in the variant stx2 prophage. The divergence of the stx2 phage in these strains strongly suggests the possibility that two separate stx2 phage acquisition events may have contributed to the emergence of these strains (Figure 8). stx2 phage can exhibit wide genetic diversity [77] and highly mosaic phage genome structures suggestive of frequent recombination between phage variants [70], [78]. While the origins of these particular stx2 phages are not clear, their inducibility is similar to that of other phage previously reported in stx2 phage-containing *E. coli* strains, and therefore these and related phages may be exchanging freely in the environment. The discovery of differences in the lysogenic stx2 phage between these strains may provide a clue to the high apparent pathogenicity of the 2011 outbreak strain, which is supported by the recent demonstration of higher inducibility of the Stx2 toxins and mRNA of the 2011 strains relative to O157:H7 strains in the presence of antibiotics [79]. While the effect on toxin production of each of the lysogenic stx2 phage in each of the isolates is not clear at this time, a previous study by Wagner and co-workers of the effect of phage genotype on toxin production using isogenic host strains harboring diverse stx2 phage yielded a broad range of toxin production levels. These differences were especially evident in uninduced (antibiotic-free) cultures [80].

While the clinical profile of the 2011 and 2009 strains appears similar, the acquisition of the IncI1 plasmid containing a broad-spectrum cephalosporinase differentiates the 2011 strains from the 2009 and HUSEC041 strains [29], [37], [64]. The mobility of these plasmids and their worldwide distribution highlight the concern over the acquisition of multi-drug resistance determinants by highly pathogenic strains. A very similar CTX-M-15-positive IncI1 plasmid was recently discovered in an isolate of *Shigella sonnei* in 2006 [81]. The presence of circulating CTX-M-15-containing plasmids worldwide [82], as well as other, even broader-spectrum beta-lactamase enzymes such as *bla*NDM-1 [83], [84], offers ample opportunity for the acquisition of this or similar multidrug-resistant plasmids to enter previously sensitive O104:H4 strains. In addition to the diversity in plasmid content, we also observed differences in the prophage content of the 2009 and 2011 outbreak isolates. While the exact roles of the divergent prophages in these isolates are not clear, several studies indicate that phage lysogeny can affect phenotypes of host strains in unexpected ways [85], [86], [87], [88]. Notably, the divergent phages between the Georgian and German isolates harbor different phage-encoded virulence factors, particularly of the *ail/lom/bor* family. These may contributed in unexpected ways to the phenotype(s) of these pathogens *in vivo* although their exact roles if any are not known at this time. Finally, questions remain about the infection mode and relative virulence of each strain. Establishment of an animal model that accurately reflects the human disease profile will be critical in future experiments for study in greater depth of this class of highly virulent *E. coli* pathogens.

Our multiphenotypic analysis revealed an unexpected trait in both of the Georgian strains, namely an increased relative resistance to polymyxin B and other membrane-disrupting agents. The basis for this resistance is not clear from the genotypes of these strains, nor is it immediately obvious whether this is a trait that was gained by the Georgian strains or lost by the 2011 isolate. While no obvious mutations (e.g. in the *phoPQ-* or *pmrAB*-regulated genes involved in regulated lipid A modification) were found in the datasets to which this phenotype could be attributed, regulation of membrane modification processes in *E. coli* is complex and highly dependent on growth conditions [89], [90]. One or more of the 2009EL–2071-specific mutations or resident prophage may contribute to this phenotype, but at this time its genetic basis remains unclear. Given that polymyxins, particularly colistins, can serve as last-line antimicrobial agents for extensively drug-resistant enterobacterial strains, particularly those that express extended-spectrum beta-lactamases, the discovery of potential emerging resistance in this highly pathogenic lineage is a concern.

Establishment and maintenance of robust global biosurveillance networks, with special emphasis on public health and disease monitoring systems and policies will be critical in the future to identify emerging disease threats such as the strains described in this study. Accurate characterization of such isolates will be increasingly important, and sequence information and comparisons can rapidly be generated both at large genome centers and using “crowd-sourcing” methodologies [38]. Finally, as the technology supporting small, more portable sequencing platforms matures, whole-genome analysis will be able to be conducted closer to the point of care during outbreaks, enabling true real-time application of genomic information to the characterization and management of ongoing disease outbreaks.

## Supporting Information

File S1 **Detailed strain phenotypes.**
(XLSX)

File S2 **Supplementary Tables S1–S5.**
(DOCX)

File S3 **Optical maps and comparison to finished sequences.**
(JPG)

File S4 **Identity of SNPs between strains and TY-2482.**
(XLSX)

File S5 **Figure showing alignments of individual prophages.**
(PDF)

File S6 **RAST annotations and three-way comparisons, including ortholog-by-ortholog analysis of Shiga toxin phage proteins.**
(XLSX)

File S7 **Omnilog data summaries (separate tabs for average AUC values for each strain; unfiltered fold-change/significance; and filtered fold-change/significance).**
(XLSX)

## References

[1] Kaper JB, Nataro JP, Mobley HL (2004) Pathogenic Escherichia coli. Nat Rev Microbiol 2: 123–140. 15040260 10.1038/nrmicro818

[2] DuPont HL (2007) The growing threat of foodborne bacterial enteropathogens of animal origin. Clin Infect Dis 45: 1353–1361. 17968835 10.1086/522662

[3] Barlow RS, Gobius KS, Desmarchelier PM (2006) Shiga toxin-producing Escherichia coli in ground beef and lamb cuts: results of a one-year study. Int J Food Microbiol 111: 1–5. 16793157 10.1016/j.ijfoodmicro.2006.04.039

[4] Berger CN, Sodha SV, Shaw RK, Griffin PM, Pink D, et al (2010) Fresh fruit and vegetables as vehicles for the transmission of human pathogens. Environ Microbiol 12: 2385–2397. 20636374 10.1111/j.1462-2920.2010.02297.x

[5] Brandl MT (2006) Fitness of human enteric pathogens on plants and implications for food safety. Annu Rev Phytopathol 44: 367–392. 16704355 10.1146/annurev.phyto.44.070505.143359

[6] Himathongkham S, Nuanualsuwan S, Riemann H, Cliver DO (2001) Reduction of Escherichia coli O157:H7 and Salmonella typhimurium in artificially contaminated alfalfa seeds and mung beans by fumigation with ammonia. J Food Prot 64: 1817–1819. 11726165 10.4315/0362-028x-64.11.1817

[7] Mootian G, Wu WH, Matthews KR (2009) Transfer of Escherichia coli O157:H7 from soil, water, and manure contaminated with low numbers of the pathogen to lettuce plants. J Food Prot 72: 2308–2312. 19903393 10.4315/0362-028x-72.11.2308

[8] van Elsas JD, Semenov AV, Costa R, Trevors JT (2011) Survival of Escherichia coli in the environment: fundamental and public health aspects. ISME J 5: 173–183. 20574458 10.1038/ismej.2010.80 PMC3105702

[9] Razzaq S (2006) Hemolytic uremic syndrome: an emerging health risk. Am Fam Physician 74: 991–996. 17002034

[10] Obrig TG (2010) Escherichia coli Shiga Toxin Mechanisms of Action in Renal Disease. Toxins (Basel) 2: 2769–2794. 21297888 10.3390/toxins2122769 PMC3032420

[11] United States Centers for Disease control and Prevention (2011) Bioterroism Agents/Diseases A to Z. Available at http://www.bt.cdc.gov/agent/agentlist.asp.

[12] Farfan MJ, Torres AG (2012) Molecular Mechanisms That Mediate Colonization of Shiga Toxin-Producing Escherichia coli Strains. Infection and Immunity 80: 903–913. 22144484 10.1128/IAI.05907-11 PMC3294676

[13] Wong ARC, Pearson JS, Bright MD, Munera D, Robinson KS, et al (2011) Enteropathogenic and enterohaemorrhagic Escherichia coli: even more subversive elements. Molecular Microbiology 80: 1420–1438. 21488979 10.1111/j.1365-2958.2011.07661.x

[14] Gyles CL (2007) Shiga toxin-producing Escherichia coli: an overview. J Anim Sci 85: E45–62. 17085726 10.2527/jas.2006-508

[15] Johnson KE, Thorpe CM, Sears CL (2006) The emerging clinical importance of non-O157 Shiga toxin-producing Escherichia coli. Clin Infect Dis 43: 1587–1595. 17109294 10.1086/509573

[16] Tyler JS, Livny J, Friedman DI (2005) Lambdoid phages and shigatoxin phages. In: Waldor MK, Friedman DI, Adhya SL, editors. Phages: Their Role in Bacterial Pathogenesis and Biotechnology. Washington, D.C.: American Society for Microbiology.

[17] Matsushiro A, Sato K, Miyamoto H, Yamamura T, Honda T (1999) Induction of prophages of enterohemorrhagic Escherichia coli O157:H7 with norfloxacin. J Bacteriol 181: 2257–2260. 10094706 10.1128/jb.181.7.2257-2260.1999 PMC93641

[18] Muhldorfer I, Hacker J, Keusch GT, Acheson DW, Tschape H, et al (1996) Regulation of the Shiga-like toxin II operon in Escherichia coli. Infect Immun 64: 495–502. 8550198 10.1128/iai.64.2.495-502.1996 PMC173792

[19] Picozzi C, Volponi G, Vigentini I, Grassi S, Foschino R (2012) Assessment of transduction of Escherichia coli Stx2-encoding phage in dairy process conditions. Int J Food Microbiol 153: 388–394. 22197444 10.1016/j.ijfoodmicro.2011.11.031

[20] Andreoli SP, Trachtman H, Acheson DW, Siegler RL, Obrig TG (2002) Hemolytic uremic syndrome: epidemiology, pathophysiology, and therapy. Pediatr Nephrol 17: 293–298. 11956886 10.1007/s00467-001-0783-0

[21] McDaniel TK, Jarvis KG, Donnenberg MS, Kaper JB (1995) A genetic locus of enterocyte effacement conserved among diverse enterobacterial pathogens. Proc Natl Acad Sci U S A 92: 1664–1668. 7878036 10.1073/pnas.92.5.1664 PMC42580

[22] Kenny B, DeVinney R, Stein M, Reinscheid DJ, Frey EA, et al (1997) Enteropathogenic E. coli (EPEC) transfers its receptor for intimate adherence into mammalian cells. Cell 91: 511–520. 9390560 10.1016/s0092-8674(00)80437-7

[23] Czeczulin JR, Balepur S, Hicks S, Phillips A, Hall R, et al (1997) Aggregative adherence fimbria II, a second fimbrial antigen mediating aggregative adherence in enteroaggregative Escherichia coli. Infect Immun 65: 4135–4145. 9317019 10.1128/iai.65.10.4135-4145.1997 PMC175595

[24] Nataro JP, Deng Y, Maneval DR, German AL, Martin WC, et al (1992) Aggregative adherence fimbriae I of enteroaggregative Escherichia coli mediate adherence to HEp-2 cells and hemagglutination of human erythrocytes. Infect Immun 60: 2297–2304. 1350273 10.1128/iai.60.6.2297-2304.1992 PMC257157

[25] Henderson IR, Nataro JP (2001) Virulence functions of autotransporter proteins. Infect Immun 69: 1231–1243. 11179284 10.1128/IAI.69.3.1231-1243.2001 PMC98013

[26] Harrington SM, Sheikh J, Henderson IR, Ruiz-Perez F, Cohen PS, et al (2009) The Pic Protease of Enteroaggregative Escherichia coli Promotes Intestinal Colonization and Growth in the Presence of Mucin. Infection and Immunity 77: 2465–2473. 19349428 10.1128/IAI.01494-08 PMC2687332

[27] Navarro-Garcia F, Sears C, Eslava C, Cravioto A, Nataro JP (1999) Cytoskeletal effects induced by pet, the serine protease enterotoxin of enteroaggregative Escherichia coli. Infect Immun 67: 2184–2192. 10225873 10.1128/iai.67.5.2184-2192.1999 PMC115956

[28] Mellmann A, Bielaszewska M, Kock R, Friedrich AW, Fruth A, et al (2008) Analysis of collection of hemolytic uremic syndrome-associated enterohemorrhagic Escherichia coli. Emerg Infect Dis 14: 1287–1290. 18680658 10.3201/eid1408.071082 PMC2600372

[29] Mellmann A, Harmsen D, Cummings CA, Zentz EB, Leopold SR, et al (2011) Prospective Genomic Characterization of the German Enterohemorrhagic Escherichia coli O104:H4 Outbreak by Rapid Next Generation Sequencing Technology. PLoS One 6: e22751. 21799941 10.1371/journal.pone.0022751 PMC3140518

[30] Scheutz F, Nielsen EM, Frimodt-Moller J, Boisen N, Morabito S, et al.. (2011) Characteristics of the enteroaggregative Shiga toxin/verotoxin-producing Escherichia coli O104:H4 strain causing the outbreak of haemolytic uraemic syndrome in Germany, May to June 2011. Euro Surveill 16. 10.2807/ese.16.24.19889-en 21699770

[31] Beutin L, Hammerl JA, Strauch E, Reetz J, Dieckmann R, et al.. (2012) Spread of a distinct Stx2-encoding phage prototype among E. coli O104:H4 strains from outbreaks in Germany, Norway and Georgia. Journal of Virology. 10.1128/JVI.00986-12 PMC3457275 22811533

[32] Bielaszewska M, Mellmann A, Zhang W, Kock R, Fruth A, et al (2011) Characterisation of the Escherichia coli strain associated with an outbreak of haemolytic uraemic syndrome in Germany, 2011: a microbiological study. Lancet Infect Dis 11: 671–676. 21703928 10.1016/S1473-3099(11)70165-7

[33] Gault G, Weill FX, Mariani-Kurkdjian P, Jourdan-da Silva N, King L, et al.. (2011) Outbreak of haemolytic uraemic syndrome and bloody diarrhoea due to Escherichia coli O104:H4, south-west France, June 2011. Euro Surveill 16. 10.2807/ese.16.26.19905-en 21749817

[34] Organization WH (2011) Outbreaks of E. coli O104:H4 infection: Update 30.

[35] European Food Safety Authority (2011) Tracing seeds, in particular fenugreek (*Trigonella foenom-graecum*) seeds, in relation to the Shiga toxin-producing E. coli (STEC) O104:H4 2011 Outbreaks in Germany and France. Parma, Italy: European Food Safety Authority. 23 p.

[36] Brzuszkiewicz E, Thurmer A, Schuldes J, Leimbach A, Liesegang H, et al.. (2011) Genome sequence analyses of two isolates from the recent Escherichia coli outbreak in Germany reveal the emergence of a new pathotype: Entero-Aggregative-Haemorrhagic Escherichia coli (EAHEC). Arch Microbiol. 10.1007/s00203-011-0725-6 PMC3219860 21713444

[37] Rasko DA, Webster DR, Sahl JW, Bashir A, Boisen N, et al (2011) Origins of the E. coli strain causing an outbreak of hemolytic-uremic syndrome in Germany. N Engl J Med 365: 709–717. 21793740 10.1056/NEJMoa1106920 PMC3168948

[38] Rohde H, Qin J, Cui Y, Li D, Loman NJ, et al (2011) Open-source genomic analysis of Shiga-toxin-producing E. coli O104:H4. N Engl J Med 365: 718–724. 21793736 10.1056/NEJMoa1107643

[39] Mossoro C, Glaziou P, Yassibanda S, Lan NT, Bekondi C, et al (2002) Chronic diarrhea, hemorrhagic colitis, and hemolytic-uremic syndrome associated with HEp-2 adherent Escherichia coli in adults infected with human immunodeficiency virus in Bangui, Central African Republic. J Clin Microbiol 40: 3086–3088. 12149388 10.1128/JCM.40.8.3086-3088.2002 PMC120615

[40] Bennett S (2004) Solexa Ltd. Pharmacogenomics 5: 433–438. 15165179 10.1517/14622416.5.4.433

[41] Margulies M, Egholm M, Altman WE, Attiya S, Bader JS, et al (2005) Genome sequencing in microfabricated high-density picolitre reactors. Nature 437: 376–380. 16056220 10.1038/nature03959 PMC1464427

[42] Zerbino DR, Birney E (2008) Velvet: algorithms for de novo short read assembly using de Bruijn graphs. Genome Res 18: 821–829. 18349386 10.1101/gr.074492.107 PMC2336801

[43] Ewing B, Green P (1998) Base-calling of automated sequencer traces using phred. II. Error probabilities. Genome Res 8: 186–194. 9521922

[44] Ewing B, Hillier L, Wendl MC, Green P (1998) Base-calling of automated sequencer traces using phred. I. Accuracy assessment. Genome Res 8: 175–185. 9521921 10.1101/gr.8.3.175

[45] Han CS, Chain P. Finishing repeat regions automatically with DupFinisher. In: Arabnia HR, Valafar H, editors; 2006. CSREA Press. 141–146.

[46] Gordon D, Abajian C, Green P (1998) Consed: a graphical tool for sequence finishing. Genome Res 8: 195–202. 9521923 10.1101/gr.8.3.195

[47] Chain PS, Grafham DV, Fulton RS, Fitzgerald MG, Hostetler J, et al (2009) Genomics. Genome project standards in a new era of sequencing. Science 326: 236–237. 19815760 10.1126/science.1180614 PMC3854948

[48] Delcher AL, Phillippy A, Carlton J, Salzberg SL (2002) Fast algorithms for large-scale genome alignment and comparison. Nucleic Acids Res 30: 2478–2483. 12034836 10.1093/nar/30.11.2478 PMC117189

[49] Kurtz S, Phillippy A, Delcher AL, Smoot M, Shumway M, et al (2004) Versatile and open software for comparing large genomes. Genome Biol 5: R12. 14759262 10.1186/gb-2004-5-2-r12 PMC395750

[50] Enright AJ, Van Dongen S, Ouzounis CA (2002) An efficient algorithm for large-scale detection of protein families. Nucleic Acids Research 30: 1575–1584. 11917018 10.1093/nar/30.7.1575 PMC101833

[51] Katoh K, Misawa K, Kuma K, Miyata T (2002) MAFFT: a novel method for rapid multiple sequence alignment based on fast Fourier transform. Nucleic Acids Res 30: 3059–3066. 12136088 10.1093/nar/gkf436 PMC135756

[52] Stamatakis A (2006) RAxML-VI-HPC: maximum likelihood-based phylogenetic analyses with thousands of taxa and mixed models. Bioinformatics 22: 2688–2690. 16928733 10.1093/bioinformatics/btl446

[53] Price MN, Dehal PS, Arkin AP (2010) FastTree 2–approximately maximum-likelihood trees for large alignments. PLoS One 5: e9490. 20224823 10.1371/journal.pone.0009490 PMC2835736

[54] Carver T, Berriman M, Tivey A, Patel C, Bohme U, et al (2008) Artemis and ACT: viewing, annotating and comparing sequences stored in a relational database. Bioinformatics 24: 2672–2676. 18845581 10.1093/bioinformatics/btn529 PMC2606163

[55] Stewart AC, Osborne B, Read TD (2009) DIYA: a bacterial annotation pipeline for any genomics lab. Bioinformatics 25: 962–963. 19254921 10.1093/bioinformatics/btp097 PMC2660880

[56] Fouts DE (2006) Phage_Finder: automated identification and classification of prophage regions in complete bacterial genome sequences. Nucleic Acids Res 34: 5839–5851. 17062630 10.1093/nar/gkl732 PMC1635311

[57] Aziz RK, Bartels D, Best AA, DeJongh M, Disz T, et al (2008) The RAST Server: rapid annotations using subsystems technology. BMC Genomics 9: 75. 18261238 10.1186/1471-2164-9-75 PMC2265698

[58] Altschul SF, Gish W, Miller W, Myers EW, Lipman DJ (1990) Basic local alignment search tool. J Mol Biol 215: 403–410. 2231712 10.1016/S0022-2836(05)80360-2

[59] Chang WE, Sarver K, Higgs BW, Read TD, Nolan NM, et al (2011) PheMaDB: a solution for storage, retrieval, and analysis of high throughput phenotype data. BMC Bioinformatics 12: 109. 21507258 10.1186/1471-2105-12-109 PMC3097161

[60] Team RDC (2009) R: A language and environment for statistical computing. Vienna, Austria: R Foundation for Statistical Computing.

[61] Crossman L (2011) Large scale expansion of mobile elements in specific hotspot regions of the German outbreak Escherichia coli O104:H4. Nature Preceedings.

[62] Miquel S, Peyretaillade E, Claret L, de Vallee A, Dossat C, et al.. (2010) Complete genome sequence of Crohn’s disease-associated adherent-invasive E. coli strain LF82. PLoS One 5. 10.1371/journal.pone.0012714 PMC2941450 20862302

[63] Parkhill J, Dougan G, James KD, Thomson NR, Pickard D, et al (2001) Complete genome sequence of a multiple drug resistant Salmonella enterica serovar Typhi CT18. Nature 413: 848–852. 11677608 10.1038/35101607

[64] Künne C, Billion A, Mshana SE, Schmiedel J, Domann E, et al (2012) Complete Sequences of Plasmids from the Hemolytic-Uremic Syndrome-Associated Escherichia coli Strain HUSEC41. Journal of Bacteriology 194: 532–533. 22207742 10.1128/JB.06368-11 PMC3256666

[65] Grad YH, Lipsitch M, Feldgarden M, Arachchi HM, Cerqueira GC, et al.. (2012) Genomic epidemiology of the Escherichia coli O104:H4 outbreaks in Europe, 2011. Proc Natl Acad Sci U S A. 10.1073/pnas.1121491109 PMC3286951 22315421

[66] Jackson SA, Kotewicz ML, Patel IR, Lacher DW, Gangiredla J, et al.. (2011) Rapid Genomic-Scale Analysis of Escherichia coli O104:H4 Using High-Resolution Alternative Methods to Next Generation Sequencing. Applied and Environmental Microbiology. 10.1128/AEM.07464-11 PMC3294476 22210216

[67] Hommais F, Krin E, Coppee JY, Lacroix C, Yeramian E, et al (2004) GadE (YhiE): a novel activator involved in the response to acid environment in Escherichia coli. Microbiology 150: 61–72. 14702398 10.1099/mic.0.26659-0

[68] Ma Z, Gong S, Richard H, Tucker DL, Conway T, et al (2003) GadE (YhiE) activates glutamate decarboxylase-dependent acid resistance in Escherichia coli K-12. Mol Microbiol 49: 1309–1320. 12940989 10.1046/j.1365-2958.2003.03633.x

[69] Yew WS, Gerlt JA (2002) Utilization of l-Ascorbate by Escherichia coli K-12: Assignments of Functions to Products of the yjf-sga and yia-sgb Operons. Journal of Bacteriology 184: 302–306. 11741871 10.1128/JB.184.1.302-306.2002 PMC134747

[70] Smith DL, Rooks DJ, Fogg PC, Darby AC, Thomson NR, et al (2012) Comparative genomics of Shiga toxin encoding bacteriophages. BMC Genomics 13: 311. 22799768 10.1186/1471-2164-13-311 PMC3430580

[71] Heffernan EJ, Wu L, Louie J, Okamoto S, Fierer J, et al (1994) Specificity of the complement resistance and cell association phenotypes encoded by the outer membrane protein genes rck from Salmonella typhimurium and ail from Yersinia enterocolitica. Infection and Immunity 62: 5183–5186. 7927803 10.1128/iai.62.11.5183-5186.1994 PMC303245

[72] Barondess JJ, Beckwith J (1995) bor gene of phage lambda, involved in serum resistance, encodes a widely conserved outer membrane lipoprotein. Journal of Bacteriology 177: 1247–1253. 7868598 10.1128/jb.177.5.1247-1253.1995 PMC176730

[73] Barondess JJ, Beckwfth J (1990) A bacterial virulence determinant encoded by lysogenic coliphage [lambda]. Nature 346: 871–874. 2144037 10.1038/346871a0

[74] Muniesa M, de Simon M, Prats G, Ferrer D, Pañella H, et al (2003) Shiga Toxin 2-Converting Bacteriophages Associated with Clonal Variability in Escherichia coli O157:H7 Strains of Human Origin Isolated from a Single Outbreak. Infection and Immunity 71: 4554–4562. 12874335 10.1128/IAI.71.8.4554-4562.2003 PMC166033

[75] Rump LV, Bodeis-Jones S, Abbott J, Zhao S, Kase J, et al.. (2011) Genetic Characterization of Escherichia coli O104 Isolates from Different Sources in the United States. Applied and Environmental Microbiology. 10.1128/AEM.07533-11 PMC3294489 22210209

[76] Brussow H, Canchaya C, Hardt WD (2004) Phages and the evolution of bacterial pathogens: from genomic rearrangements to lysogenic conversion. Microbiol Mol Biol Rev 68: 560–602. 15353570 10.1128/MMBR.68.3.560-602.2004 PMC515249

[77] Smith DL, Wareing BM, Fogg PCM, Riley LM, Spencer M, et al (2007) Multilocus Characterization Scheme for Shiga Toxin-Encoding Bacteriophages. Applied and Environmental Microbiology 73: 8032–8040. 17951439 10.1128/AEM.01278-07 PMC2168134

[78] Johansen BK, Wasteson Y, Granum PE, Brynestad S (2001) Mosaic structure of Shiga-toxin-2-encoding phages isolated from Escherichia coli O157:H7 indicates frequent gene exchange between lambdoid phage genomes. Microbiology 147: 1929–1936. 11429469 10.1099/00221287-147-7-1929

[79] Laing CR, Zhang Y, Gilmour MW, Allen V, Johnson R, et al (2012) A Comparison of Shiga-Toxin 2 Bacteriophage from Classical Enterohemorrhagic Escherichia coli Serotypes and the German E. coli O104:H4 Outbreak Strain. PLoS One 7: e37362. 22649523 10.1371/journal.pone.0037362 PMC3359367

[80] Wagner PL, Acheson DWK, Waldor MK (1999) Isogenic Lysogens of Diverse Shiga Toxin 2-Encoding Bacteriophages Produce Markedly Different Amounts of Shiga Toxin. Infection and Immunity 67: 6710–6714. 10569798 10.1128/iai.67.12.6710-6714.1999 PMC97090

[81] Folster JP, Pecic G, Krueger A, Rickert R, Burger K, et al (2010) Identification and Characterization of CTX-M-Producing Shigella Isolates in the United States. Antimicrobial Agents and Chemotherapy 54: 2269–2270. 20211893 10.1128/AAC.00039-10 PMC2863668

[82] Bush K, Fisher JF (2011) Epidemiological expansion, structural studies, and clinical challenges of new beta-lactamases from gram-negative bacteria. Annu Rev Microbiol 65: 455–478. 21740228 10.1146/annurev-micro-090110-102911

[83] Poirel L, Lagrutta E, Taylor P, Pham J, Nordmann P (2010) Emergence of metallo-ss-lactamase NDM-1-producing multidrug resistant Escherichia coli in Australia. Antimicrob Agents Chemother. 10.1128/AAC.00878-10 PMC2976126 20823289

[84] Yong D, Toleman MA, Giske CG, Cho HS, Sundman K, et al (2009) Characterization of a new metallo-beta-lactamase gene, bla(NDM-1), and a novel erythromycin esterase gene carried on a unique genetic structure in Klebsiella pneumoniae sequence type 14 from India. Antimicrob Agents Chemother 53: 5046–5054. 19770275 10.1128/AAC.00774-09 PMC2786356

[85] Godeke J, Paul K, Lassak J, Thormann KM (2011) Phage-induced lysis enhances biofilm formation in Shewanella oneidensis MR-1. ISME J 5: 613–626. 20962878 10.1038/ismej.2010.153 PMC3105746

[86] Loeffler JM, Fischetti VA (2006) Lysogeny of Streptococcus pneumoniae with MM1 Phage: Improved Adherence and Other Phenotypic Changes. Infection and Immunity 74: 4486–4495. 16861634 10.1128/IAI.00020-06 PMC1539626

[87] Schuch R, Fischetti VA (2009) The secret life of the anthrax agent Bacillus anthracis: bacteriophage-mediated ecological adaptations. PLoS One 4: e6532. 19672290 10.1371/journal.pone.0006532 PMC2716549

[88] Chen Y, Golding I, Sawai S, Guo L, Cox EC (2005) Population fitness and the regulation of Escherichia coli genes by bacterial viruses. PLoS Biol 3: e229. 15984911 10.1371/journal.pbio.0030229 PMC1151598

[89] Gibbons HS, Kalb SR, Cotter RJ, Raetz CR (2005) Role of Mg2+ and pH in the modification of Salmonella lipid A after endocytosis by macrophage tumour cells. Mol Microbiol 55: 425–440. 15659161 10.1111/j.1365-2958.2004.04409.x

[90] Kus JV, Gebremedhin A, Dang V, Tran S-L, Serbanescu A, et al (2011) Bile Salts Induce Resistance to Polymyxin in Enterohemorrhagic Escherichia coliO157:H7. Journal of Bacteriology 193: 4509–4515. 21725004 10.1128/JB.00200-11 PMC3165498

