# Supplementary material for: Genomic Comparison of Escherichia coli O104:H4 Isolates from 2009 and 2011 Reveals Plasmid, and Prophage Heterogeneity, Including Shiga Toxin Encoding Phage stx2
Source: PLoS One. 2012 Nov 1;7(11):e48228. doi: 10.1371/journal.pone.0048228 (PMC3486847; doi:10.1371/journal.pone.0048228)
Supplement: File S2 — Supplementary Tables S1–S5. (DOCX) [file pone.0048228.s002.docx]

**Supplementary Data File S2: Summary of whole-genome sequencing data generated for each *E. coli* strain**

**Table S1: WGS Dataset Descriptions**

| **Project** | **Illumina** | | **454 Titanium** | | | |
| --- | --- | --- | --- | --- | --- | --- |
|  | Reads | Total Data | Shotgun Reads | PE Ave Insert | PE Reads | Total Data |
| 2011C-3493 | 68,001,128 | 6,800.1 Mb | 190,316 | 7 kb | 48,226 | 85.4 Mb |
| 2009EL-2050 | 64,810,939 | 6,481.1 Mb | 1,134,847 | 7 kb | 196,203 | 479.7 Mb |
| 2009EL-2071 | 57,082,874 | 5,708.3 Mb | 1,229,574 | 9 kb | 295,352 | 379.7 Mb |

**Table S2: Draft sequencing data included in the final assemblies**

| **Project** | **454 Draft Data Used** | **454 Data Genome Coverage** | **Illumina Data Used** | **Illumina Data Genome Coverage** |
| --- | --- | --- | --- | --- |
| 2011C-3493 | 84.1 Mb | 16.2x | 1,590 Mb | 305.8x |
| 2009EL-2050 | 170.6 Mb | 32.8x | 6,481.1 Mb | 1,246.4x |
| 2009EL-2071 | 181.1 Mb | 34.8x | 5,708.3 Mb | 1,097.7x |

**Table S3: NCBI Accession Numbers for genomes used in phylogenetic analysis**

| AE005174 | Escherichia coli O157-H7 EDL933 |
| --- | --- |
| AE005674 | Shigella flexneri 2a 301 |
| AE014073 | Shigella flexneri 2a 2457T |
| AE014075 | Escherichia coli CFT073 |
| AM946981 | Escherichia coli BL21 DE3 |
| AP009048 | Escherichia coli K-12 |
| AP009240 | Escherichia coli SE11 |
| AP009378 | Escherichia coli SE15 |
| AP010953 | Escherichia coli 11368 |
| AP010958 | Escherichia coli 12009 |
| AP010960 | Escherichia coli 11128 |
| AP012030 | Escherichia coli DH1 |
| AP012306 | Escherichia coli K-12 |
| BA000007 | Escherichia coli Sakai |
| CP000034 | Shigella dysenteriae 1 Sd197 |
| CP000036 | Shigella boydii 4 Sb227 |
| CP000038 | Shigella sonnei Ss046 |
| CP000243 | Escherichia coli UTI89 |
| CP000247 | Escherichia coli 536 |
| CP000266 | Shigella flexneri 5 8401 |
| CP000468 | Escherichia coli APEC O1 |
| CP000800 | Escherichia coli E24377A |
| CP000802 | Escherichia coli HS |
| CP000819 | Escherichia coli REL606 |
| CP000946 | Escherichia coli ATCC 8739 |
| CP000948 | Escherichia coli K-12 |
| CP000970 | Escherichia coli SMS-3-5 |
| CP001063 | Shigella boydii BS512 CDC 3083-94 |
| CP001164 | Escherichia coli EC4115 |
| CP001368 | Escherichia coli O157-H7 TW14359 |
| CP001383 | Shigella flexneri Fxv 2002017 |
| CP001396 | Escherichia coli K-12 |
| CP001509 | Escherichia coli BL21 DE3 |
| CP001637 | Escherichia coli DH1 |
| CP001665 | Escherichia coli BL21-Gold DE3pLysS AG |
| CP001671 | Escherichia coli ABU 83972 |
| CP001846 | Escherichia coli O55-H7 CB9615 |
| CP001855 | Escherichia coli O83-H1 NRG 857C |
| CP001969 | Escherichia coli IHE3034 |
| CP002167 | Escherichia coli UM146 |
| CP002185 | Escherichia coli W |
| CP002211 | Escherichia coli clone D i2 |
| CP002212 | Escherichia coli clone D i14 |
| CP002516 | Escherichia coli KO11 |
| CP002729 | Escherichia coli UMNK88 |
| CP002797 | Escherichia coli NA114 |
| CP003034 | Escherichia coli O7-K1-NM CE10 |
| CU928145 | Escherichia coli 55989 |
| CU928160 | Escherichia coli IAI1 |
| CU928161 | Escherichia coli S88 |
| CU928162 | Escherichia coli ED1a |
| CU928163 | Escherichia coli UMN026 |
| CU928164 | Escherichia coli IAI39 |
| FM180568 | Escherichia coli O127-H6 E2348-69 |
| FN554766 | Escherichia coli 042 |
| FN649414 | Escherichia coli ETEC H10407 |
| U00096 | Escherichia coli K-12 |
| CU928158 | Escherichia fergusonii ATCC 35469T |

**Table S4: Chromosome and Plasmid statistics –**

|  | **Strain** | | | |
| --- | --- | --- | --- | --- |
|  | **TY2482** | **2011C-3493** | **2009EL-2050** | **2009EL-2071** |
| **Chromosome** |  |  |  |  |
| NCBI Accession # |  | CP003289 | CP003297 | CP003301 |
| Total Length | 5278900 | 5273097 | 5253138 | 5312586 |
| # CDS | 5127 | 5205 | 5174 | 5254 |
| % coding sequence | 87.18 | 87.44 | 87.38 | 87.29 |
| G+C content (%) |  |  |  |  |
| Total genome (%) | 50.72 | 50.71 | 50.74 | 50.72 |
| Protein coding genes (%) | 50.58 | 50.53 | 50.60 | 5054 |
| RNA genes | 58.31 | 58.14 | 58.22 | 58.24 |
| Ribosomal RNA |  |  |  |  |
| # 16S loci | 7 | 7 | 7 | 7 |
| # 23S loci | 7 | 7 | 7 | 7 |
| # 5S loci | 8 | 8 | 8 | 8 |
| # tRNA loci | 101 | 92 | 92 | 95 |
| **Plasmid #1** | **pESBL** | **pESBL-EA11** | **N/A** | **N/A** |
| Accession number |  | CP003290 |  |  |
| Total Length | 88695 | 88544 |  |  |
| # CDS | 115 | 90 |  |  |
| % coding sequence | 87.43 | 77.00 |  |  |
| G + C content (%) |  |  |  |  |
| Total replicon (%) | 49.72 | 49.73 |  |  |
| Protein coding genes (%) | 49.54 | 49.73 |  |  |
| ^1^Est. copy # | N/A | 1 |  |  |
| **Plasmid #2** | **pAA** | **pAA-EA11** | **pAA-09EL50** | **pAA-09EL71** |
| Accession number |  | CP003291 | CP003298 | CP003302 |
| Total Length | 75330 | 74217 | 74213 | 75573 |
| # CDS | 122 | 86 | 86 | 89 |
| % coding sequence | 82.27 | 72.27 | 70.57 | 72.16 |
| G + C content (%) |  |  |  |  |
| Total replicon (%) | 47.23 | 47.09 | 47.08 | 47.17 |
| Protein coding genes (%) | 47.24 | 49.01 | 49.06 | 49.16 |
| Est. copy # | N/A | 1 | 1 | 1 |
| **Plasmid #3** | **pG2011** | **pG-EA11** | **pG-09EL50** | **pG-09EL71** |
| Accession number |  | CP003292 | CP003299 | CP003303 |
| Total Length | 1549 | 1549 | 1549 | 1549 |
| # CDS | 1 | 1 | 1 | 1 |
| % coding sequence | 30.79 | 30.79 | 30.79 | 30.79 |
| G + C content (%) |  |  |  |  |
| Total replicon (%) | 50.81 | 50.81 | 50.81 | 50.81 |
| Protein coding genes (%) | 42.77 | 42.77 | 42.77 | 42.77 |
| Est Copy # |  | 26 | 3 | 1 |
| **Plasmid #4** | **N/A** | **N/A** | **p09EL50** | **N/A** |
| Accession number |  |  | CP003300 |  |
| Total Length |  |  | 109274 |  |
| # CDS |  |  | 112 |  |
| % coding sequence |  |  | 74.52 |  |
| G + C content (%) |  |  |  |  |
| Total replicon (%) |  |  | 45.31 |  |
| Protein coding genes (%) |  |  | 45.68 |  |
| RNA genes (%) |  |  | 53.12 |  |
| Ribosomal RNA |  |  |  |  |
| # tRNA loci |  |  | 2 |  |
| Est. Copy # |  |  | 1 |  |

^1^Copy number estimated by dividing the average sequence coverage of the replicon by the average coverage of the chromosome

**Table S5: Prophage Attachment (attB) sites**

| **Phage** | **Att Site** |
| --- | --- |
| A-Black | ND |
| B-Pink | TTGCAGGTTCGATTCC |
| C-Grey | ND |
| D-Orange | ND |
| E-Green | TCTTCACGCTTA |
| F-Blue | ND |
| G-Red | ACCGCCTGCTTT |
| H-Yellow | GGCTTTTGGCGT |
| I-Purple | TCAGCCGGACAG |
| J-Grey | N.D |
| 55989-1 | TTGCAGGTTCGATTCC |
| 55989-2 | N.D |
| 55989-3 | AAACCAGAAAA |
| 55989-4 | TCAGCCGGACAG |
| 55989-5 | CGGTCTTGAAAACCGGCGACCCGAAAGGGTTCCAGAGTTCGAATCTCTGCGCTTCCGCCAAA |
| 55989-6 | GGCTTTTGGCGT |
