# Supplementary figures and images for: Genomic Comparison of Escherichia coli O104:H4 Isolates from 2009 and 2011 Reveals Plasmid, and Prophage Heterogeneity, Including Shiga Toxin Encoding Phage stx2
Source: PLoS One. 2012 Nov 1;7(11):e48228. doi: 10.1371/journal.pone.0048228 (PMC3486847; doi:10.1371/journal.pone.0048228)

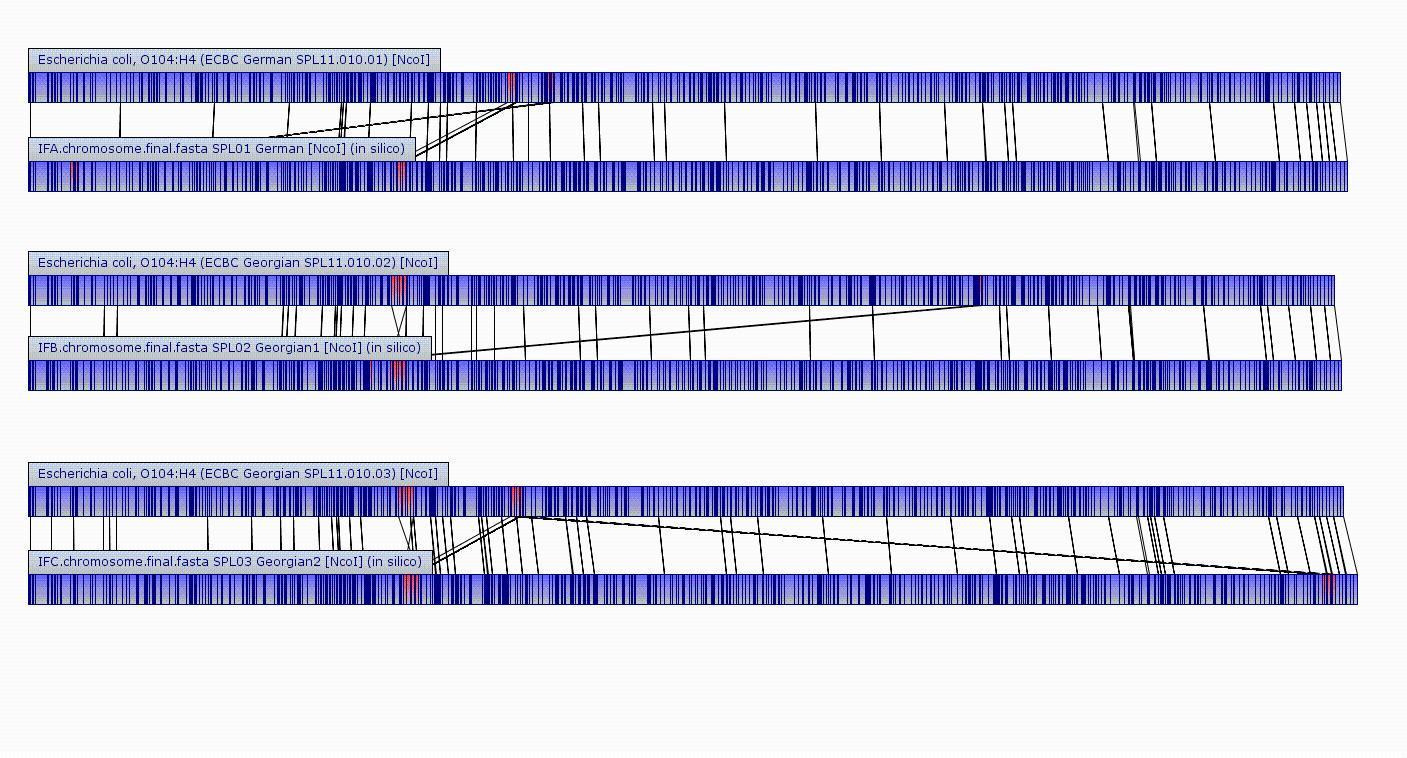

Supplement: File S3 — Optical maps and comparison to finished sequences. (JPG) [file pone.0048228.s003.jpg]
