# Supplementary material for: Genomic Comparison of Escherichia coli O104:H4 Isolates from 2009 and 2011 Reveals Plasmid, and Prophage Heterogeneity, Including Shiga Toxin Encoding Phage stx2
Source: PLoS One. 2012 Nov 1;7(11):e48228. doi: 10.1371/journal.pone.0048228 (PMC3486847; doi:10.1371/journal.pone.0048228)
Supplement: File S5 — Figure showing alignments of individual prophages. (PDF) [file pone.0048228.s005.pdf]

Supplemental File S5

Phage A (Black)

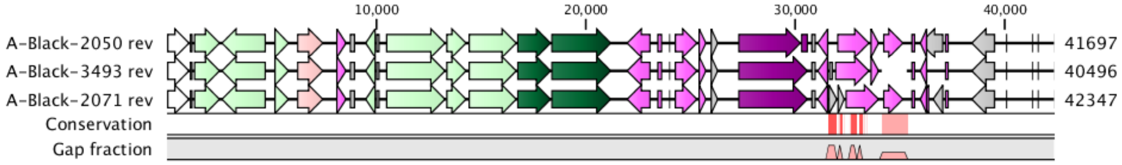

Phages C/I (Grey/Purple)

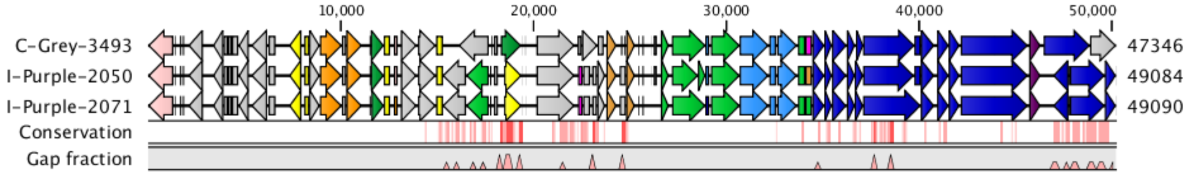

Phage D (Orange)

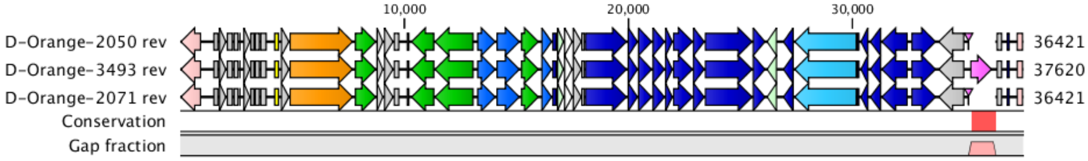

Phage E (Green)

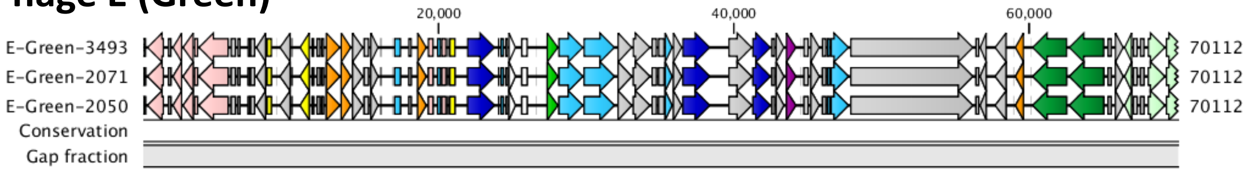

Phage F (Blue)

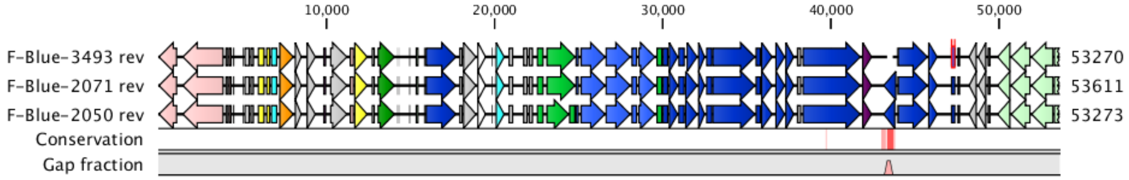

Phage H (Yellow)

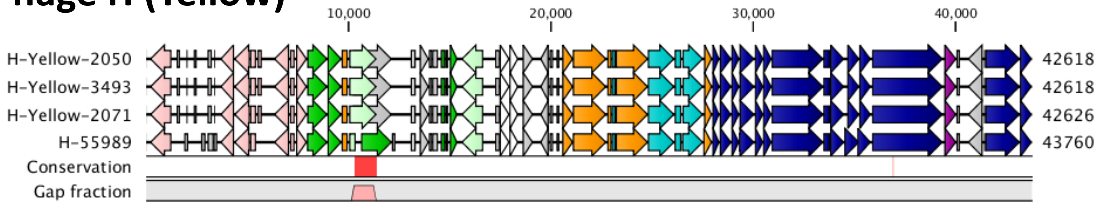

Figure S5 – Alignment of individual prophage sequences. Prophage sequences were aligned using the multiple alignment tool in CLC Bio. Divergent regions are indicated by red bars, while gaps are indicated by the line graph; the height of the line graph indicates the number of sequences that contain a gap.
